# Advanced Optical Wavefront Technologies to Improve Patient Quality of Vision and Meet Clinical Requests

**DOI:** 10.3390/polym14235321

**Published:** 2022-12-05

**Authors:** Martina Vacalebre, Renato Frison, Carmelo Corsaro, Fortunato Neri, Sabrina Conoci, Elena Anastasi, Maria Cristina Curatolo, Enza Fazio

**Affiliations:** 1 Dipartimento di Scienze Matematiche ed Informatiche, Scienze Fisiche e Scienze della Terra (MIFT), Università di Messina, V.le Ferdinando Stagno d’Alcontres 31, 98166 Messina, Italy; 2 Optical Consultant SIFI SpA, 95025 Aci Sant’Antonio (CT), Italy; 3 Dipartimento di Scienze Chimiche, Biologiche, Farmalogiche ed Ambientali (CHIBIOFARAM), Università di Messina, V.le Ferdinando Stagno d’Alcontres 31, 98166 Messina, Italy; 4 Innovation and Medical Science, SIFI SpA, 95025 Aci Sant’Antonio (CT), Italy

**Keywords:** wavefront sensing, adaptive sensors, optical aberrations, Shack–Hartmann sensor, wavefront-guided laser refractive surgery, ophthalmology

## Abstract

Adaptive optics (AO) is employed for the continuous measurement and correction of ocular aberrations. Human eye refractive errors (lower-order aberrations such as myopia and astigmatism) are corrected with contact lenses and excimer laser surgery. Under twilight vision conditions, when the pupil of the human eye dilates to 5–7 mm in diameter, higher-order aberrations affect the visual acuity. The combined use of wavefront (WF) technology and AO systems allows the pre-operative evaluation of refractive surgical procedures to compensate for the higher-order optical aberrations of the human eye, guiding the surgeon in choosing the procedure parameters. Here, we report a brief history of AO, starting from the description of the Shack–Hartmann method, which allowed the first in vivo measurement of the eye’s wave aberration, the wavefront sensing technologies (WSTs), and their principles. Then, the limitations of the ocular wavefront ascribed to the IOL polymeric materials and design, as well as future perspectives on improving patient vision quality and meeting clinical requests, are described.

## 1. Introduction

In the second half of the 20th century, ophthalmologists started to investigate how to reduce the dependence on spectacles by means of refractive surgery [1]. These eye disorders were treated by using an excimer laser to modify the shape of the cornea and therefore its refractive state. Despite the success of the refractive surgery, patients complained about glare, halos, and starburst in both day and night vision [2,3]. The clinical data showed that higher-order aberrations (HOAs) were induced by laser refractive surgery [2]. Hence, it was necessary to expand a new area of research, known today as “wavefront technology”, which aimed to measure and reduce the induction of these unwanted aberrations [4].

Wavefront technology is largely applied in astronomy to correct aberrations in the reflecting mirrors of telescopes to obtain images with higher quality, as in the case of the Hubble Space Telescope and NASA’s James Webb Space Telescope, which has recently been providing very high-quality images of space [5,6]. Nowadays, an analogous approach is used for the wavefront-guided refractive surgery: an aberrometer was introduced in the procedure to collect the eye’s wavefront errors in order to guide the excimer laser on a customized profile [2]. The knowledge and the tools obtained for this purpose had a great impact on other aspects of ophthalmology such as corrective devices (both contact and intraocular lenses) and in the evaluation of the progress of eye diseases [4,7]. 

Specifically, in wavefront-guided (WFG) laser-assisted in situ keratomileusis (LASIK), also named custom LASIK, an aberrometer provides complex patterns that will be ablated by the laser. There are many variables considered in order to obtain an optimal outcome in terms of optical ablation, among them are the choice of the patient, the acquisition of high-quality wavefront data, and the execution of a successful surgery towards the precise prediction and control of the eventual variations happening during healing. Hence, WFG treatments based on the high-resolution aberrometer promote not only a visual improvement but also allow refractive predictability and a post-operative level of higher-order aberrations (HOAs) [8]. In particular, topography-integrated wavefront-guided (TI-WFG) LASIK is a new approach for calculating the ablation profile based on the combination of the topography and ocular wavefront aberrations obtained with a previously validated high-resolution topographer–aberrometer [9].

Firstly, this review aims to summarize the theoretical principles concerning wavefront science and the main devices used to measure aberrations, taking into account the properties of lens materials. For example, most wavefront-sensing devices used in conjunction with an excimer laser employ a Hartmann–Shack sensor. As described below in detail, this device divides the light exiting the eye into a grid pattern for analysis by a lenslet array. Other techniques use ray tracing analysis (spatially resolved refractometry), a retinal imaging concept (Tscherning method), or dynamic skiascopy (time-based aberrometry using an infrared slit scan of the optical system). In the second part of this work, the main clinical applications of the technology are reviewed: refractive surgery, the lens materials, their design, and the adaptive optics. The advantages and the limitations ascribed to each one are outlined. Particular attention will be paid to intraocular lenses (IOLs). IOLs represent the most advanced solution for cataract refractive surgery and the correction of astigmatism (a type of refractive error due to rotational asymmetry in the eye’s refractive power) and presbyopia (natural loss of the ability to focus on nearby objects that occurs with ageing). Nowadays, the refractive cataract is at the frontier of cataract surgery as it combines clouded lens removal with the simultaneous correction of refractive errors, such as myopia, hypermetropia, astigmatism, and presbyopia, in order to free the patient from the need to wear glasses following the surgery. The most advanced IOLs for this purpose are the EDOF (extended depth of focus) lenses that present an optical plate with a continuous series of focuses to ensure a continuum correction from far to near in the case of presbyopia and, in the case of astigmatism, to provide compensation for the corneal abnormal curvature.

Recent devices have provided more realistic ways to mimic accommodation by simulating natural optical aberrations and the image formation at the retina, but there are still many challenges to overcome. To this end, the laboratory evaluation of higher-order aberrations and light scattering in IOLs (mainly opacified) must be still carried out. Specifically, an alternative methodology using a new optical setup equipped with WF sensors should be designed and fine-tuned to characterize the quality of the new generation of IOLs. 

Our overview aims to present the wavefront technologies widely adopted to date and also to highlight their limitations when applied to multifocal IOLs, which are widely requested by the international market. This topic is not particularly dealt with in other reviews [3,4,7,10,11,12] and requires our peculiarity and originality. As the IOL trend is oriented towards lenses of increasing complexity, it is necessary to have wavefront analysis instrumentation that is, in turn, able to follow the complexity of the lens to allow its validation, compliance with the optical design, quality control, and consistency with production batches. This need forces the development trend towards the wavefront in AO technology by, for example, not using Shack–Hartmann or Talbot Moiré sensors but moving towards pyramid or curvature sensors.

## 2. Wavefront Analysis Based on Zernike Polynomials

As is known, a wavefront is an imaginary surface representing the ensemble of points of a propagating radiation characterized by the same phase. In our case, once light rays refract when passing across the lens and cornea, a wavefront forms on the surface end of the emanating rays. The size of the entrance pupil determines the X-Y extension of the wavefront, being the same, whereas the Z dimension, marking a deviation from the optical path, corresponds to the aberration of the eye. Therefore, if no aberrations are present, the light rays emanating from the eye would be parallel, thus creating a flat wavefront. In fact, an eye is optically perfect when the wavefront shape is a sphere centered at infinity, which also allows the elimination of some focusing errors. Therefore, in such a case, we deal with a plane wavefront indeed formed by the light rays reflected back from the perfect emmetropic eye. 

**Figure 1:**
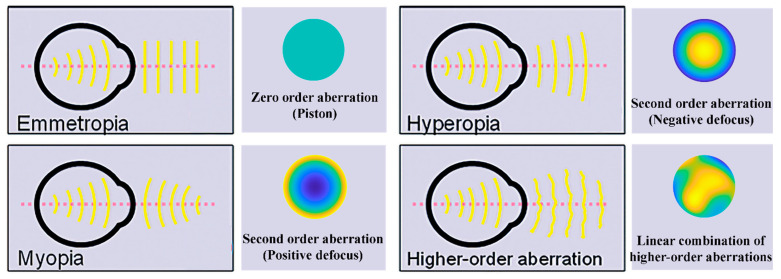
Scheme of wavefronts and corresponding contour maps for different cases. Teal color indicates no deviations; colors towards yellow indicate positive deviations, while those towards blue indicate negative deviations. Therefore, in the case of the myopic eye (bottom-left panel) the wavefront is represented by a paraboloid with the minimum in the center and the maximum at the edge. The contrary happens in the case of the hyperopic eye (top-right panel): the paraboloid is turned upside down with the maximum in the center and the minimum at the edges. Figure adapted with permission from Wiley from Ref. [3] Naoyuki Maeda, Clinical applications of wavefront aberrometry—a review, *Clinical & Experimental Ophthalmology*, 37: 118–129. © 2009 Royal Australian and New Zealand College of Ophthalmologists.

Concerning eye diseases, myopic eyes, being characterized by converging rays, produce a concave wavefront. Conversely, hyperopic eyes are characterized by emanating divergent rays, thus creating a convex wavefront. Finally, astigmatic eyes have a wavefront which is saddle-shaped due to an elliptic-shaped cornea [13,14]. It is noteworthy that the aberrations of higher orders produce wavefronts whose shape complexity increases with the aberration order itself. In the presence of aberrations, the image is defocused or distorted since the rays do not converge anymore to a unique image point [4]. The difference between the actual wavefront and the ideal reference is called the wavefront error or wavefront aberration [3]. It is qualitatively represented by a contour map, where the points characterized by the same amount of aberration share the same color [4], as depicted in Figure 1.

A quantitative insight is given in terms of the root mean square (RMS) of the wavefront deformation, defined as the root square of the wavefront variance. The RMS is equal to zero in an ideal case and assumes a positive value in an aberrated wavefront [15,16]. The wavefront data are decomposed in a linear sum of terms thanks to Zernike polynomials [13], revealing the total root mean square (RMS) error. The combination of Zernike independent functions is suitable for representing complex surfaces in terms of polar coordinates (r, θ) [4], as reported below:

(1) $$W\left(r,\theta \right)=\underset{n,m}{\sum }C_{n}^{m}Z_{n}^{m}\left(r,\theta \right),$$

**Figure 2:**
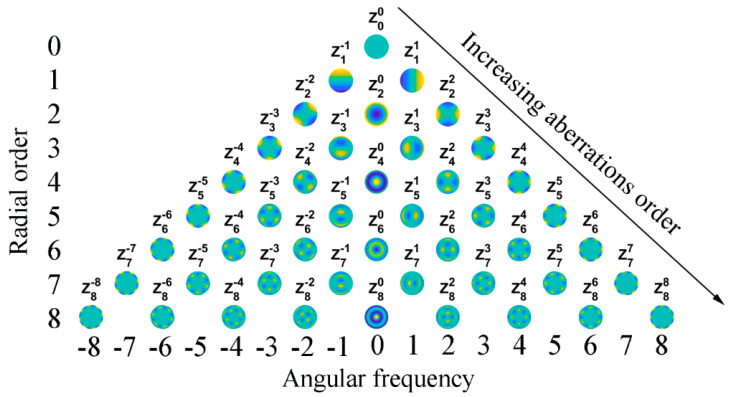
Zernike pyramid. As for previous figure colors towards yellow indicate positive deviations, while those towards blue indicate negative deviations. Zero deviations are indicated in teal color.

The coefficient *C_n_^m^* is proportional to the weight of a specific Zernike aberration present in the system. The subscript *n* is known as the radial order and assumes only positive values, whereas the superscript *m* is the angular frequency and can be either positive or negative [4,13]. Figure 2 shows the so-called Zernike pyramid, which is useful in describing the ordering system for Zernike polynomials. Commonly, when analyzing aberrometry data for normal and abnormal eyes, the 0th radial order coefficient (called a piston) and the 1st radial order coefficients (called a ‘tip’ and a ‘tilt’) are usually ignored because they refer only to a phase shift or to an image displacement, respectively, and not to its quality. Instead, the 2nd order terms are related to defocus and astigmatism optical aberrations, whereas some of the 3rd and 4th orders are, respectively, related to the primary spherical aberration ($Z_{4}^{0}$) and coma aberrations ($Z_{3}^{-1}$ to vertical coma and $Z_{3}^{1}$ to horizontal coma).

On increasing the radial order, we move towards terms corresponding to higher-order aberrations, which are characterized by a more complex shape and terminology. The description of the effect of the eye’s optical properties on the image quality can be performed by approaches involving image-plane metrics [17]. Among them are the point spread function (PSF), representing the image quality for point objects, and the optical transfer function (OTF), which is used for grating objects. As reported below in Equations (2) and (3), the PSF is the squared modulus of the Fourier transform of the pupil function P(x,y), wherein A(x,y) describes the amplitude distribution and W (x,y) the wavefront deformation on the considered pupil [18,19]. The OTF can be obtained as the inverse Fourier transform of the point spread function (Equation (4)) [19].

(2) $$P\left(x,y\right)=A\;\left(x,y\right)\;e^{i\;\frac{2\pi }{\lambda }\;W\left(x,y\right)}$$

(3) $$PSF\;\left(x,y\right)={\left|FT\;\left[P\left(x,y\right)\right]\right|}^{2}$$

(4) $$OTF\;\left(\xi \;,\eta \right)=FT^{-1}\;\left[PSF\left(\xi ,\eta \right)\right]$$

Image-plane metrics describe the wavefront error in the plane of the retina. Furthermore, the aberrations can affect the image of a grating by reducing the contrast or translating the image sideways to create a spatial phase shift. The changes with the spatial frequency of the image contrast and phase shift are, respectively, described by the modulation transfer function (MTF) and the phase transfer function (PTF), which are expressed, respectively, by the real and imaginary contribution of the OTF (referred to as the OTF modulus and phase—see Equations (5) and (6)).

(5) MTF (ξ,η)=Re[OTF(ξ,η)]

(6) PTF (ξ,η)=Im[OTF (ξ,η)]

In this context, despite the mentioned limitations, the wavefront aberrometry has been applied in clinical ophthalmology to heal eye diseases and, in particular, for designing wavefront-guided refractive surgery. However, there is still space for improvement, especially in the field of wavefront sensors to accurately measure higher-order aberrations.

## 3. Wavefront Sensors for Ophthalmological Applications: Physical Principle and Practice

Wavefront sensors can be defined as aberrometers, revealing light wave distortion after it passes via the eye’s optical system. On the market, various wavefront sensing devices employing different technologies can be found [20,21,22]. The most widely used wavefront sensors, including the Shack–Hartmann sensor, the pyramidal prism, and the Tscherning aberrometer, are reviewed in the following, and their advantages and drawbacks are briefly summarized in Table 1. Some technical details found in the literature, such as dynamic range and sensitivity, are reported in Table 2 and compared with the Shack–Hartmann wavefront sensor.

Generally, wavefront sensors can be classified in two groups: outgoing and ingoing. The former covers the techniques in which the light source is set on the retina, and the wavefront coming out from the eye is studied. An example of an outgoing sensor is the Shack–Hartmann. Conversely, the ingoing aberrometers are focused on the alterations present in the wavefront after it went through the eye, e.g., the Tscherning aberrometer and the ray-tracing system [23]. Another difference between the several wavefront sensors is in the target. Some sensors, such as the Shack–Hartmann or pyramid sensors, measure the first derivative of the wavefront phase (WF), while other kinds of sensors, such as the curvature ones, aim to measure the second derivative of the WF phase.

**Table 1:** Wavefront sensors for ophthalmological applications.

	Description	Advantage	Drawback
**Shack–Hartmann WS**	Detection of spot displacements thanks to a lenslet array and a reference grid.	Flexibility and adaptability to different measurement systems [24].	Limited dynamic range [15] and limited spatial sampling [25]. Not suitable for high-resolution phase measurement [26]. Cost [24].
**Pyramid Sensor**	A pyramid prism divides the incoming light into four different spots on a CCD surface. Their differences provide information about WF gradients.	Adjustable sampling and dynamic range [27].	Spurious reflections, necessity of another device for modulation [27].
**Curvature Sensor**	Two detectors are symmetrically placed with respect to the focal plane. Their difference in intensity provides information about the second derivative of the WF.	Compared to S–H, higher dynamic range and lower cost [15].	Long measurement time and less accurate for higher-order aberrations [15].
**Optical Differentiation WS**	A system of lenses and a mask is used to obtain WF phase slope thanks to Fourier transform properties.	Achromaticity, high resolution, and large dynamic range [28].	Lost absorbed energy [29]. Compared to S–H, worse signal-to-noise ratio [29].
**Diffuser WS**	A thin diffuser is set close to the detector and its memory effect is used to retrieve WF displacements.	Low cost, large dynamic range [24].	Slow computational time [30].
**Shearing Interferometry**	The interference pattern between the incoming wavefront and its displaced replica is used to measure the wavefront phase.	No need for a reference wave [15]	Limited dynamicrange [31].
**Tscherning Aberrometer**	A collimated laser beam illuminates a mask with regular matrix pin holes, forming a bundle of thin parallel rays. The deviations of all spots from their ideal regular positions are associated with the optical aberrations, computed in the form of Zernike polynomials up to the 8th order.	Fast measuring and highly accurate.	Not patient friendly because it requires more time and effort to obtain treatable image [32].

**Table 2:** Comparison between Shack–Hartmann WS and other wavefront sensors in terms of dynamic range and sensitivity. A plus sign + is used when better working ranges than those of the Shack–Hartmann WS have been reported in the literature for the sensor under consideration; in the opposite case, a minus sign − is used. For Shack–Hartmann WS, numerical working ranges are added as a reference from a study published by McKay et al. (Ref. [24]).

	Dynamic Range	Sensitivity
Shack–Hartmann WS	−4 D to 4.5 D [24]	0.13 D [24]
Pyramid Sensor	− [15]	+ [33]
Curvature Sensor	+ [15]	− [15]
Optical Differentiation WS	+ [28]	− [28]
Diffuser WS	+ [24]	+ [24]
Multiple Shearing Interferometry	+ [34]	+ [34]
Talbot Moiré Interferometry	+ [35]	Not available
Tscherning Aberrometer	+ [36]	Not available

### 3.1. Shack–Hartmann Sensor

**Figure 3:**
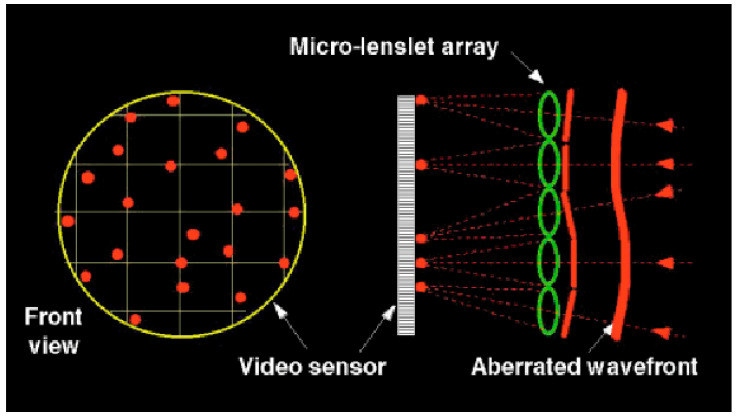
Working principle of the Shack–Hartmann sensor: the micro-lenslet array (green ellipses) creates spots (full red circles) on the sensor according to the wavefront (big solid red line) coming out of the eye. Figure reused with permission from Ref. [47] Larry N. Thibos, Principles of Hartmann-Shack Aberrometry, *Journal of Refractive Surgery* 16, S563-S565, 2000, copyright International Society of Refractive Surgery.

The Shack–Hartmann (S–H) sensor [37,38] is the most used wavefront sensor in astronomy and ophthalmology [39]. Originally, it was developed for military purposes to fulfil the need for improved images from satellites [40]. As depicted in Figure 3, the sensor is made of a lenslet array creating spots from the incident light, whose spatial displacements from a reference grid registered on a CCD camera are a direct measure of the wavefront tilts [15,41]. Specifically, the distance of each spot from its ideal position is measured and related to the local distortions in the pupil due to the optics of the eye. Its main drawbacks are the cost and the limited dynamic range due to the used lenslet array. Some studies were published on the possibility of expanding the S–H sensor’s dynamic range [11]. Shinto et al. proposed an adaptive spot search method based on a dual microlens array and confirmed the dynamic range expansion for defocus, astigmatism, and coma [42]. More recently, Akondi and Dubra presented an algorithm to improve the lenslet image location in the cases of defocus and astigmatism [43]. The individual spot displacement allows the computation of the local slope of the wavefront over each lenslet aperture; as a consequence, the S–H sensor does not take into account the quality of the individual spots formed by the lenslet array, and it is particularly inaccurate for highly aberrated eyes. In fact, if the wavefront shape within a single lenslet varies significantly, the spot pattern formed by that lenslet can be blurred, thus reducing the maximum wavefront slope that can be measured reliably. The blurring of the lenslet focal spot of the Hartmann–Shack sensor can be partly neglected by taking into account that the center of such blurred focal distribution obeys the laws of geometric optics, analogously with the case of the classical and quantum description of a particle [44,45,46]. A further limitation of the S–H sensor is due to the lenslet spacing (number of lenslets across the pupil) and the lenslet array focal length. Note that the majority of the higher-order aberrations are typically included in Zernike modes up to 8th order Zernike coefficients, corresponding to 42 coefficients in total (see Figure 2), indicating that at least 42 lenslets are needed to measure higher-order aberrations (HOAs). The distance within a lenslet’s subaperture (corresponding to one-half of the lenslet’s diameter) is the maximum displacement that each spot can perform on the used CCD camera.

To overcome these limitations, different approaches have been used. The projection of a tight and well-defined spot onto the eye retina (achieved by restricting the illuminating beam diameter) is one of the approaches adopted to analyze eyes with highly aberrated corneal optics. This requires a larger CCD camera to capture the spot array pattern. S–H-based sensors are still widely employed, as is the laser ray-tracing (R-T) technique. The latter, developed in 1997, consists of a thin-diameter beam of light, which is projected onto the retina sequentially, and the distance to the retinal reference position is used to calculate the specific aberrations of the eye. Ultimately, the main difference between the S–H and the R-T sensors is the methodology used to acquire the spot image. In the laser R-T technique, the incident beam is scanned sequentially over the entrance pupil to measure light going into the eye, while the S–H sensor measures light coming out of the eye. In this case, a parallel process is necessary to acquire multiple spots over the exit pupil. The sequential acquisition of wavefront aberrations has the advantage of avoiding the possibility of ‘overlapping’ optical phenomena, whereas simultaneous acquisition measurements need short acquisition times to achieve a high accuracy in assessing wavefront error [48].

Recently, Wu et al. proposed a modification of the S–H sensor by replacing the lenslet array with a spatial light modulator (SLM) in order to provide a multi-megapixel resolution [49]. By combining a CMOS sensor with a phase-retrieval algorithm, they obtained a higher spatial resolution (one order of magnitude) than that in the current non-interferometric WF sensors [49].

### 3.2. Foucault Knife-Edge and Optical Differentiation Wavefront Sensor (ODWS)

The Foucault knife-edge test and the linear amplitude filter are techniques of spatial filtering. The spatial filtering techniques operate on an image, taking into consideration the intensity values in a suitable neighborhood of each pixel. Linear filtering is one of the most powerful image-enhancement methods. It is a process in which part of the signal frequency spectrum is modified by the transfer function of the filter. In general, the filters under consideration are linear and shift-invariant; so, the output images are characterized by the convolution sum between the input image and the filter impulse response.

The Foucault knife-edge test was described in 1858 by French physicist Léon Foucault as a way to measure the conic shapes of optical mirrors. In the Foucault knife-edge test, a spherical surface, a point source, and a knife edge are used to evaluate the possible transversal aberrations. Specifically, a knife edge is placed near the focus and passed through the image of a point or slit source. The shadow, observed by the eye (shown) or on a screen, gives information about the aberration content. A perfect lens will have one image point that darkens almost instantaneously when the knife edge passes though the image. These shadow patterns are based on geometrical analysis, while diffraction will blur out the edges. The variations of these shadow patterns give information about the spherical surface, enabling the user to precisely determine the position of the focal point of the curved mirror [50,51,52].

**Figure 4:**
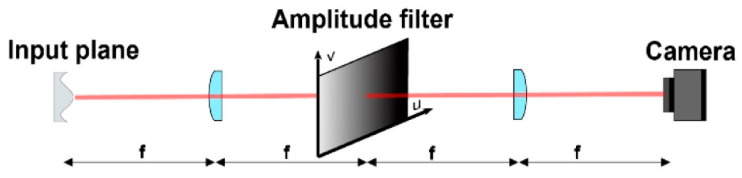
Principle of optical differentiation WF sensor. A filter with linear field transmission gradient is located at the Fourier plane of a 4f line. Figure reused from Ref. [28] under the terms of the OSA Open Access Publishing Agreement, © 2019 Optical Society of America.

The optical differentiation wavefront sensor (ODWFS) resembles the Foucault knife-edge test principles. In fact, the working principle of the optical differentiation wavefront sensor (ODWFS) [53] consists in the insertion of a linear amplitude filter in a focal plane filtering setup. In this way, a continuous Foucault knife-edge test is carried out instead of the normal discrete knife-edge test. Furthermore, its dynamic range is very high, but its sensitivity is low. As shown in Figure 4, the setup is a telescopic system made of a first achromatic lens (L1), which performs the Fourier transform of the input; a mask with variable transmittance, used as filter (OF); a second achromatic lens, performing the inverse Fourier transform of the product of the previous elements; and a CCD surface for the photometric detection [53,54]. Thanks to the differentiation property of the Fourier transform, the detected intensity is directly linked to the WF phase derivative [53].

The more general implementation of an optical differentiation wavefront sensor (ODWS), based on several wavefront gradients obtained by amplitude modulation in a coherent filtering setup, was pioneered by Bortz [55]. It requires a spatially varying transmission filter in the far field of the source under test. So, the radiant energy received by the surface per unit area (fluence), measured in the image plane of the pupil, is related to the wavefront slope in the direction of the transmission gradient (see the optical setup in Figure 4, where a typical 4f spatial filtering system is shown). The spatially varying transmission filter is set in the Fourier plane: the combination of the propagation in a thin lens of focal length f and an additional propagation by a distance f allows the field at the input pupil to be inverse Fourier transformed to the detection plane (i.e., to the far field). We outline that, without the filter, the fluence F0(x,y) measured in that plane is identical to the input fluence, after taking into account an obvious spatial inversion.

The ODWS performance was evaluated using the twelve Zernike polynomials of radial order, defining the test wavefronts over the circular input pupil [56]. It emerges that the main advantages of this sensor are the high resolution, the possibility to use it with polychromatic source, and the large dynamic range [54]. Conversely, a great amount of energy is lost due to the absorption of the mask; so, it will impact the signal-to-noise ratio (SNR) [53,57]. Oti et al. compared the SNR between S–H sensor and the ODWS. They observed that, even in adverse conditions, the ODWS shows a better SNR than the Shack–Hartmann for high resolution sensing [53]. Furthermore, compared to most interferometric techniques, the ODWS does not have a strong coherence requirement, e.g., it can operate with non-monochromatic sources. Despite these advantages, the ODWS is not widely used due to the practical difficulty of manufacturing components with well-controlled transmission profiles.

### 3.3. Pyramid Sensor

**Figure 5:**
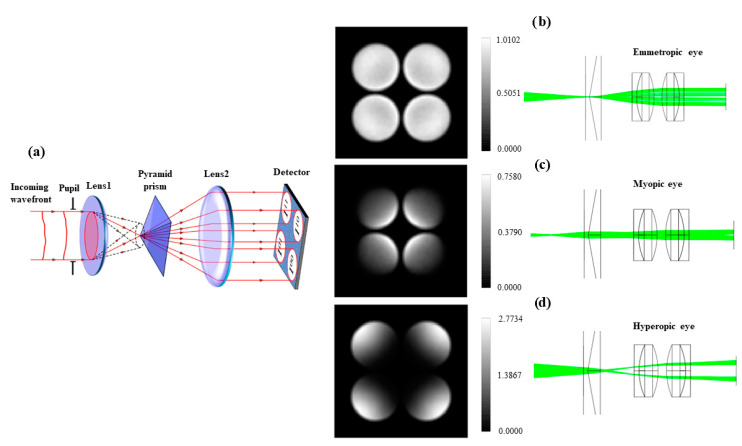
(**a**) Pyramid sensor (adapted with permission from Ref. [58] I. Shatokhina, V. Hutterer, R. Ramlau, Review on methods for wavefront reconstruction from pyramid wavefront sensor data, *Journal of Astronomical Telescopes, Instruments, and Systems*, 6 (1), 010901 (2020). © 2020 Society of Photo-Optical Instrumentation Engineers (SPIE). (**b**–**d**) Simulated CCD images of the source at different pyramid positions and Zemax images showing the ray behavior close to the PS for different aberrations. See main text for details.

Since the first implementation in 1997, adaptive optics (AO) systems for ophthalmic applications have always relied on S–H sensors to perform wavefront sensing. While this choice was obviously successful in most cases, one could also think of alternative wavefront sensing approaches to achieve this task with possibly higher efficiency and greater flexibility. The application of the pyramid sensor (PS) to ocular wavefront measurements is a valid alternative thanks to its flexibility in measuring a broad range of ocular aberrations. Similarly to the Foucault knife-edge test [50], in the pyramidal wavefront sensors (PS), the aberration-induced inhomogeneities are sensed by placing in the focal plane a four-facet pyramid refractive element with its tip aligned to the optical axis (see Figure 5a). 

The wavefront gradients along the two orthogonal directions are retrieved from the intensity distribution among the four pupil images [15]. In this way, pupil sampling and sensing sensitivity can be adjusted separately. Zemax OpticStudio software (ZEMAX LCC, Kirkland, WA, USA) was used to analyze the common optical aberrations and the contribution to image degradation across the full x-y field of view. This allows the achieving of important information to correct the considered aberration. Figure 5b reports the image of the source conjugated with the pyramid position and the corresponding simulated CCD image for an emmetropic eye. Similarly, Figure 5c,d report the images of the source projected in front of and beyond the pyramid WFS and the corresponding simulated CCD images for myopic and hyperopic eyes, respectively. 

Numerical simulations comparing the S–H and the PS suggest that the latter may operate with a higher sensitivity in closed-loop conditions [33]. We remember that the sampling parameters of a Shack–Hartmann sensor are fixed and depend on the components’ design [59]. Instead, the pyramid sensor proposed by Ragazzoni [27,59] overcomes this difficulty and allows the adjustment of the sampling parameters on the basis of the sample. The main advantages of this type of sensor are the great adaptability to different orders of aberration and the easy modification of the dynamic range [15,27,60]. In addition, the Shack–Hartmann sensors must employ some methods to average out the speckle caused by the roughness of the retina; these methods are not necessary when using the pyramid sensor, whose main disadvantage is due to the spurious reflections which can be detected from the anterior ocular surfaces [27].

### 3.4. Curvature and Phase Diversity Wavefront Sensors

**Figure 6:**
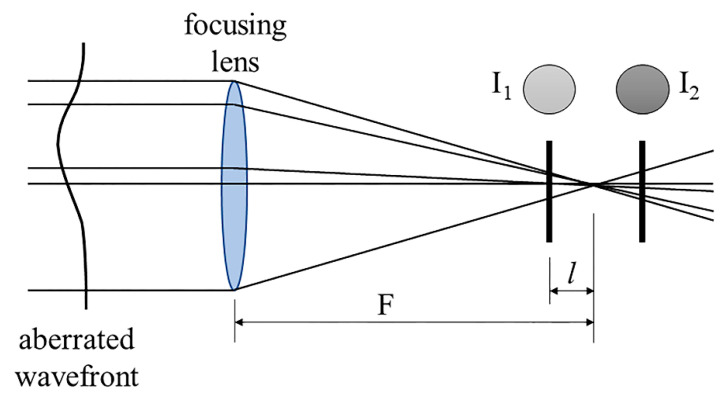
Principle of the curvature wavefront sensor. The wavefront curvature distribution can be evaluated by measuring the non-uniform illumination at two positions, before and after the pupil plane. Figure reprinted from Ref. [15] New methods and techniques for sensing the wave aberrations of human eyes, M. Lombardo and G. Lombardo, *Clinical and Experimental Optometry*, 2009 Taylor & Francis Ltd., with permission of the publisher (Taylor & Francis Ltd., Abingdon-on-Thames, UK, http://www.tandfonline.com).

In 1988, Roddier proposed a new method in the wavefront analysis, namely the curvature sensor (CS). The principle is based on the reconstruction of the second derivative of the wavefront. As shown in Figure 6, two detectors are set at a certain distance *l* from the focal plane. The distance *l* is directly proportional to the spatial resolution and inversely proportional to the sensitivity [41]. In some cases, a vibrating mirror at the lens focus provides the modulation of the sampled positions, and the wavefront sensor synchronously measures the modulated signal. The difference in light intensity distributions between the two planes is used to evaluate the local WF aberrations [41]. Several algorithms, such as the Green function and the Gureyev–Nugent algorithm, can be used [15]. In 2006, Díaz-Doutón et al. adapted a CS for an ocular aberration measurement for the first time and obtained a similar performance to that of the S–H sensor [61]. Similarly, Torti et al. [62] investigated the feasibility of using a curvature sensor in the ophthalmic field and evidenced that, as compared to S–H sensor, the curvature WF sensor was not limited anymore by the features of the lenslet array, showing a larger dynamic range [15,62]. However, it is fundamental to find a good trade-off, which requires a prolonged time of computing. A large defocus is needed to measure the wavefront with higher resolution, thus reducing the sensitivity of the sensor [15]. 

Curvature sensors are similar to the phase diversity wavefront sensor (PDWS). The PDWS simultaneously records two images, one in the focal plane and another, known as the “diverse image”, in a defocused plane. As with all the other curvature sensors, both images are taken in out-of-focus planes [63], and the wavefront is connected to intensity via propagation physics. The main useful features of the PDWS as exploited in ophthalmic applications are: (1) it provides a real image of the pupil; (2) it accommodates variability in iris location, size, and shape, but it may be critical to resolve the phase on speckled beams; (3) it allows equally spaced sample planes with equal magnification of all images; and (4) it simplifies the sensor alignment, calibration, and data processing. Finally, the dynamic range and wavefront sensitivity are controlled by sample plane spacing and camera digitization bit depth; unlike the SHWS, they are not coupled to the spatial resolution. Ultimately, the PDWS works like the diffractive IOL multi-plane imaging, allowing the easy analysis of complex optical systems.

### 3.5. Diffuser Wavefront Sensor

**Figure 7:**
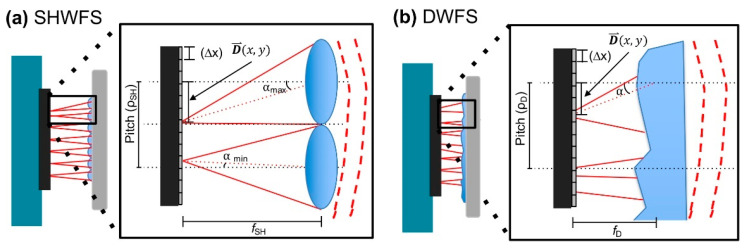
Differences between the dynamic range explored by SHWFS (**a**) and DWFS (**b**). The big squares represent the zoomed vision of the selected area. Figure reused from Ref. [24] under the terms of the OSA Open Access Publishing Agreement, © 2019 Optical Society of America.

Many efforts have been made over time to find low-cost alternatives to common wavefront sensors. The possibility to use a thin diffuser and its memory effect is promising. The principle is based on the correspondence between a tip/tilt in an incoming wavefront and the corresponding local shift in the detected pattern [30]. The diffuser is set close to the camera and the wavefront is reconstructed numerically by a specific algorithm [30]. Berto et al. proposed the use of the known “Demon Algorithm” [64], which has been optimized to perform the non-rigid registration of bio-medical images [30]. A weak diffuser at distinct angles of illumination has been adopted by Gunjala et al. to reconstruct the aberration profiles from multiple images by a statistical approach [65]. Recently, McKay et al. have developed a diffuser wavefront sensor (DWFS) for ocular aberrometry with a larger dynamic range with respect to a Shack–Hartmann wavefront sensor (SHWFS) for wavefront measurements [24] (Figure 7).

**Figure 8:**
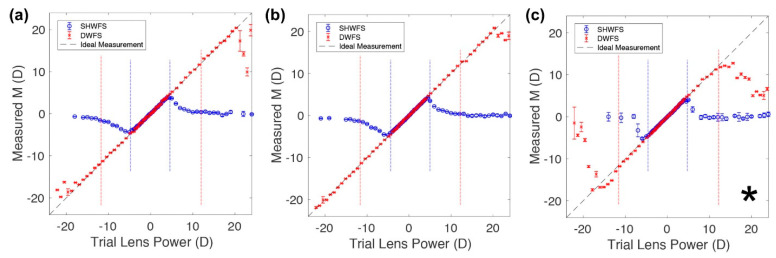
The measured spherical equivalent power (M) versus the trial lens power for SHWFS (blue symbols) and DWFS (red symbols) for three different illuminating sources: LD (**a**), LD+LSR (**b**), and LED (**c**). Dashed vertical lines indicate the corresponding predicted dynamic range. * Power-limited. Figure reused from Ref. [24] under the terms of the OSA Open Access Publishing Agreement, © 2019 Optical Society of America.

This is clear by looking at Figure 7a and recalling some of the geometrical concepts. In fact, in the case of the SHWFS, the α angle, defined by the incoming wavefront, determines the spot displacement on the CCD, which in turns also depends on the lenslet pitch (ρ_SH_) and focal length (f_SH_). The pixel size Δx corresponds to the lowest detectable spot deviation, whereas ρ_SH_/2 limits its detectable maximum value. Note that the deviation amount of each spot for a given wavefront tilt scales with f_SH_ for tan(α) ≃ α [66]. Finally, these geometrical constraints, together with the other properties such as the signal-to-noise ratio and the spot-tracking algorithm, define the SHWFS dynamic range and sensitivity with respect to the angular wavefront tilt (α_max_ and α_min_). Considering the DWFS (Figure 7b), McKay et al. used non-periodic lenslet arrays in which the diffuser pitch (ρD), evaluated to be 338 +/− 21 µm, corresponds to the mean distance between the sharp caustic intensity bands and the diffuser focal length (fD), empirically chosen to be equal to 5.15 mm, to the distance from the diffuser to the sensor [24]. By using trial lenses with spherical power in the range of [−24 D, +24 D], located close to the model eye lens, it has been possible to evaluate the dynamic range of both types of sensors. Figure 8 reports the measurements of the spherical equivalent power (M) in both the SHWFS (blue symbols) and the DWFS (red symbols) for three different illuminating sources: a laser diode (LD, Figure 8a); an LD with a laser speckle reducer (LD+LSR, Figure 8b); and (3) a light-emitting diode (LED, Figure 8c). 

Although the limited power of the LED (marked by an asterisk) did not allow precise measurements, the DWFS showed a larger dynamic range than that of the SHWFS for all the illuminating sources. This is also testified to by the dashed vertical lines shown in Figure 8 corresponding to the predicted dynamic range [24].

### 3.6. Shearing Interferometry

A Mach Zehnder interferometer and an optically addressed spatial light modulator (OASLM) has been adopted as a novel adaptive wavefront correction system [67]. In this system, the output fringe intensity from the interferometric element is fed back optically to the OASLM, which is placed in one arm of the interferometer. In this way, a real-time correction of aberrated wavefronts, without electronic calculations, was obtained by a reliable reconstruction of the eye’s wavefront aberration (WA) achieved by the interferometric element.

The shearing interferometry is the most used technique for optical tests. The recorded interferometry is between the incoming wavefront and its displaced replica [15]. No reference wave is necessary since, starting from the modification, three different methods can be obtained: “lateral shear”, if the input is shifted; “radial shear”, if it is magnified; and “rotational shear”, if it is rotated [15]. For all these shearing interferometric sensors, the phase information collected is proportional to the gradient of the test wavefront in the direction of the shear. Different optical setups can be used to obtain the shearing, e.g., wedge plates, polarizing prisms, gratings, or diffractive optical elements (DOEs) [15].

**Figure 9:**
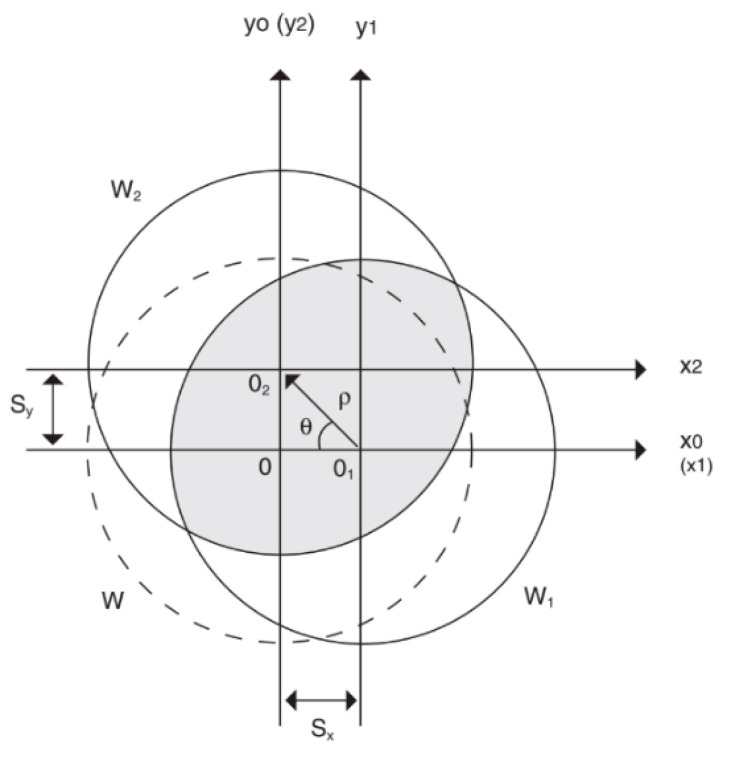
Coordinate system of the original (W, dashed circle) and the two sheared wavefronts, W1 and W2, as when adopting the shearing interferometry. Figure reprinted from Ref. [15] New methods and techniques for sensing the wave aberrations of human eyes, M. Lombardo and G. Lombardo, *Clinical and Experimental Optometry*, 2009 Taylor & Francis Ltd., with permission of the publisher (Taylor & Francis Ltd., Abingdon-on-Thames, UK, http://www.tandfonline.com).

The phase of the incoming wavefront is reconstructed by an iterative method known as “phase-shifting”. The object (e.g., the retina) is illuminated with a single beam of coherent light. In the simplest case, a grating is situated in front of the object to provide two sheared images of the object. The object is then imaged onto a CCD array sensor. A shearing device in the imaging system results in two superimposed images: the relative separation, or shearing distance, is normally chosen to be a small fraction of the field of view. Therefore, any pixel in the sensor device receives light from two points on the object surface and the phase changes at the pixel then depend directly on the relative displacement of the two points. As shown in Figure 9, the sheared wavefronts can be generated by a grating mounted on a translation stage. The interference pattern is generated in the overlap area (in a grey color) of the two sheared wavefronts. S_x_ and S_y_ are the amount of shear in the x and y directions, respectively; r is the shear vector, and q is the shear angle. 

The main disadvantage of the shearing interferometry is the limited dynamic range [31]. To overcome these limitations, ‘multiple shearing interferometry’ was recently adopted, and its principle relies on the generation of several replicas of the wavefront, which are evaluated using a conventional grating. Such interferometers are able to detect the phase distortions of several tens of waves but also of very small fractions of a wave (λ/100). At the same time, the sensitivity and dynamics can be continuously adapted to the analyzed aberrations [68].

#### Talbot Moiré Technology

**Figure 10:**
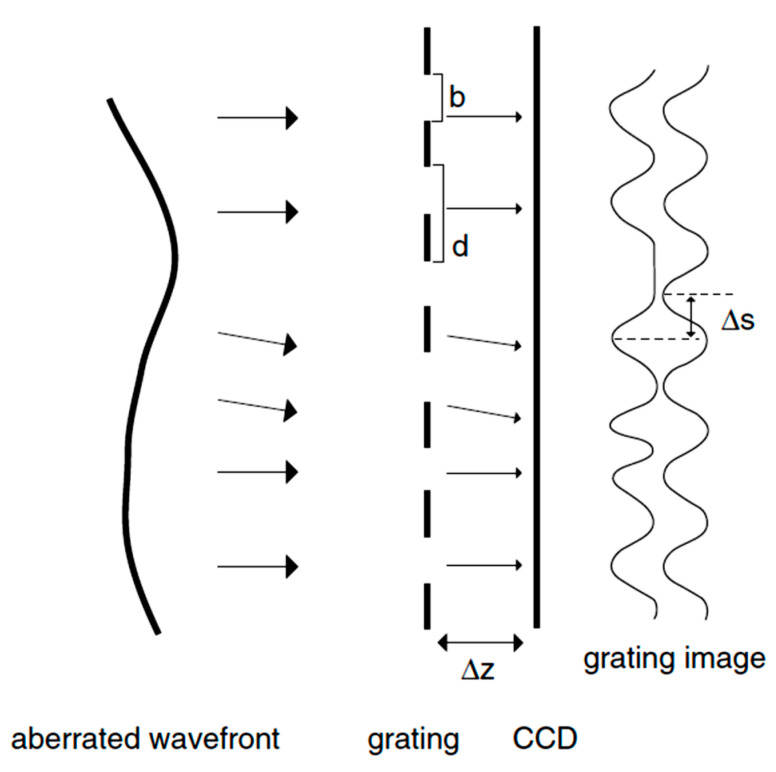
Talbot Moiré principle where d represents the spacing of the grating, b the width of the slit, Δz the Talbot distance and Δs the local phase shift of the fundamental frequency component of the grating image. Figure reprinted from Ref. [15] New methods and techniques for sensing the wave aberrations of human eyes, M. Lombardo and G. Lombardo, *Clinical and Experimental Optometry*, 2009 Taylor & Francis Ltd., with permission of the publisher (Taylor & Francis Ltd., Abingdon-on-Thames, UK, http://www.tandfonline.com).

The Talbot interferometer belongs to the class of lateral shearing interferometers. Talbot Moirè technology can be applied to detect wavefront tilts by gratings [69], generating Moirè fringes which replicate themselves at a certain distance, known as the Talbot distance Δz, where the CCD surface is set (see Figure 10). The Talbot distance Δz is given by the relation [70]:

(7) $$\Delta z=\frac{2d^{2}}{\lambda }$$

where *d* is the period of the grating, and λ is the light wavelength. The Talbot Moiré technology uses: (1) the Talbot image of a two-dimensional grating as a wavefront sensor and (2) the local shift of the Talbot image to calculate the tilt of the wavefront. By estimating the phases of the fundamental spatial frequency between the grating and the local patch of the Talbot image, the definition of the shift of the Talbot image is allowed [15]. The Talbot Moiré sensor is constructed with two gratings, in which the Moiré fringes are generated by superimposing the Fourier image of the first grating on the second. The two gratings have the same period. If the phase object is placed in front of the first grating, the light deflected by the object yields the shifted Fourier images, and the resultant Moiré fringes show the deflection mapping [71].

The distortion of the fringe pattern reflects the local tilt of the wavefront. The diffraction patterns can be observed at specific periodic distances from the grating (called Talbot images) [72]. Sekine et al. [69] used a two-dimensional grating for sensing the optical wavefront with the CCD placed in the plane of the Talbot image of the first order to maximize the contrast of the grating image. Overall, the common advantage of the Talbot interferometry is its relatively simple and inexpensive design when compared to the other opto-electronic systems, as well as its accuracy and high spatial resolution. Furthermore, the dynamic range is larger in comparison to the Shack–Hartmann sensor [15,73]. The disadvantages of this sensor technology are the sensitivity to vibration, the changes in the polarization of the beam coming back out of the eye, and the complex reconstruction of the phase error. All these factors strongly limit the widespread application of this technology to human eyes.

### 3.7. Tscherning Aberrometer, Ray-Tracing System, and Dynamic Skiascopy

**Figure 11:**
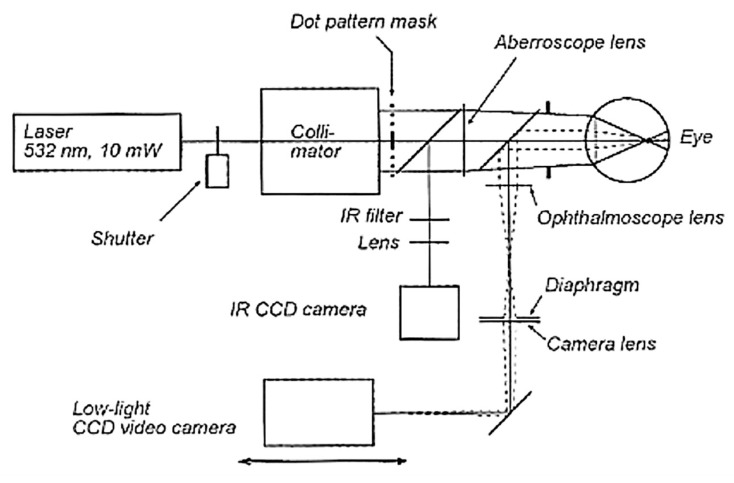
Scheme of the wavefront analyzer based on the principles of Tscherning aberrometer. Figure reused with permission from Ref. [32], M. Mrochen et al., Principles of Tscherning Aberrometry, *Journal of Refractive Surgery* 16, S570-S571, 2000, copyright International Society of Refractive Surgery.

As described in a previous section, H–S aberrometers are fairly user-friendly and offer very high resolution, reproducibility, and accuracy, as well as a quick fundamental time in their measurements and analysis of ocular aberrations. However, H–S is inadequate when reconstructing the wavefronts of patients with highly aberrated corneas. The same limitation characterizes the Tscherning aberrometers. The latter are fast-measuring and highly accurate, but they are less patient-friendly because they require more time and effort to obtain a treatable image. A scheme of the Tscherning aberrometer is shown in Figure 11. The Tscherning aberrometer uses a laser beam (generally, patients are disturbed by the green (532 nm) line used as a source) and projects a grid on the target. Any distortion from the reference grid is reported in the aberration map [26].

**Figure 12:**
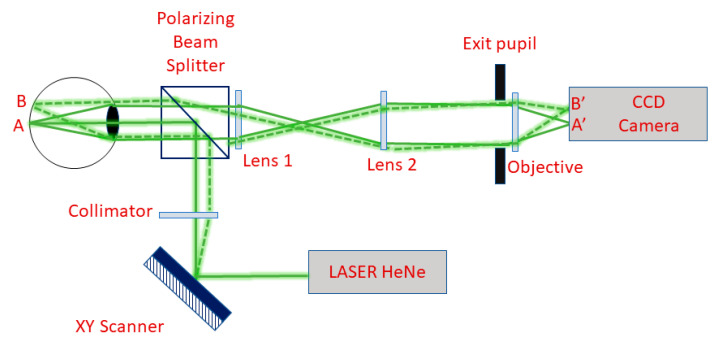
Basic setup of the laser ray tracing technique. The retinal image (A, B) is projected onto a CCD camera forming the image (A’, B’). Figure adapted with permission from Ref. [48] © The Optical Society.

Another type of aberrometry is the ray tracing, which works on a similar principle to that of the Tscherning. The main difference between them is that the ray-tracing system scans the retina sequentially instead of simultaneously. So, each point is processed separately and sequentially with the advantage that it can reduce the risk of intersecting light rays, enabling more highly aberrated eyes to be imaged. However, the ray tracing technique is limited by the resolution of the aberroscope [15]. An unexpanded laser beam is scanned so that it enters the eye sequentially through different pupil locations. One marginal ray (dotted line in Figure 12) and the principal ray (solid line) are shown. Each retinal image (A, B) is projected onto a CCD camera. The displacement of the image, with respect to a reference, is proportional to the local derivative of the wave aberration [48,74]. 

An interesting system uses the skiascopic ocular wavefront-sensing device (also named the retinoscopy technique), which is a time-dependent method (not a position-dependent approach) to study optical aberrations (mainly, the refractive error of the eye). In this case, the measurement of the time gap between the reflected light beams, thanks to a rotating array of detectors, is directly linked to the wavefront errors. The series of sensors that rotate very rapidly allows the collection of more than 1400 retinoscopic data points in a short period of time [75]. Further information is reported in the next sections, which describe some of the ophthalmological imaging methods and the applications.

## 4. IOLs Wavefront Aberrations Experimental Setups

**Figure 13:**
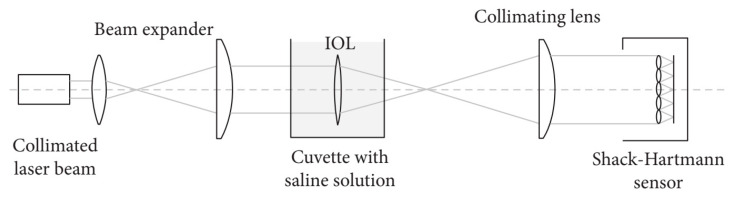
Scheme of a simple optical setup for the measurement of wavefront aberrations. Figure reprinted from Ref. [76] under the terms of the Creative Commons Attribution License.

Some optical setups are used for measuring the wavefront aberrations of the lens and specifically of the multifocal intraocular lenses (IOLs). Multifocal IOLs can be classified into two types: diffractive and refractive. Refractive IOLs have two or more curvatures to form refractive zones, whereas diffractive IOLs create more than one retinal image throughout the light diffraction. The scheme of one of the simplest setups is shown in Figure 13. The system consists of a diode-collimated laser beam of 532 nm, a beam expander, a transparent cell (filled with 0.9% normal saline solution) in which the IOL was submerged, a collimating lens, and a Shack–Hartmann wavefront sensor. An XYZ translational stage is attached to the wet cell to align the IOL with the optical axis of the wavefront sensor.

**Figure 14:**
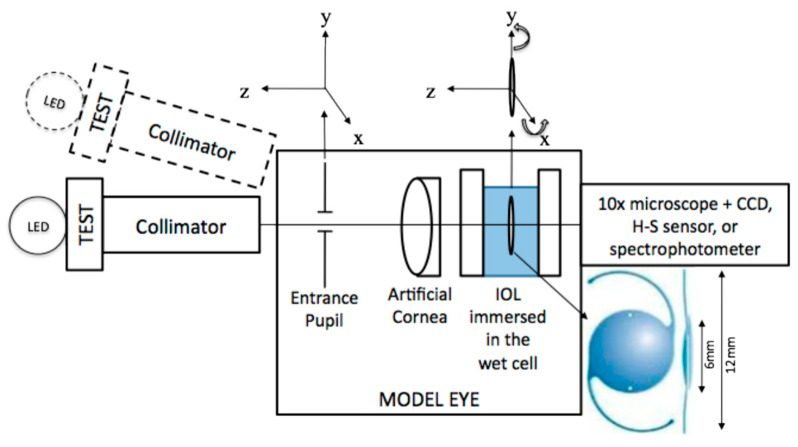
Scheme of an optical layout using an artificial cornea. Reprinted from Ref. [77] under the terms of the Creative Commons Attribution (CC BY) license by IOP Publishing Ltd., Bristol, UK.

Some sophisticated test benches [77] have been designed for the lens optical characterization, including the possibility of testing the lens under off-axis conditions as well as in the presence of decentration and/or tilt in agreement with ISO 11979-2:2014 [78]. This aspect is particularly relevant for characterizing the intraocular lens (IOL) under conditions close to a real human eye. To perform this, artificial corneas with different amounts of spherical aberrations (SA) are generally used. The main parts of the setup shown in Figure 14 are (1) the illumination sources (white lamp and four types of LED at different wavelengths in the 459–637 nm range) to study the chromatic dispersion, which is a relevant parameter in multifocal lenses; (2) a USAF test chart for detecting the image quality assessment; (3) a collimator to analyze the IOLs according to the ISO standard (the object has to be at infinity); (4) some pinholes with different diameters to check the lens optical performance; (5) the model eye with the artificial cornea; (6) the wet cell where the IOL is immersed (in some cases, a water bath containing 0.01% fluorescein solution was used to visualize the propagation of light rays illuminated by a monochromatic green laser light (532 nm) [79]); and then (7) the image and wavefront analysis (a 10× microscope and a Hartmann–Shack sensor). 

Once the experimental data are acquired, the lens optical imaging quality is assessed by using common metrics, such as the modulation transfer function (MTF), the point spread function (PSF), and/or the Strehl ratio (SR). A further analysis is the analysis of the lens’s wavefront through expansion in Zernike polynomials. This bench tests the ability of an optical system to reproduce an infinitesimally thin cross-slit image. The cross-sectional intensity profile of the reproduced image is then calculated into MTF values via the Fourier transform of the line spread function. A similar setup has been used to estimate the energy distribution between the CCD collected images as a function of pupil diameter. The authors found that, for large pupils, the energy efficiency of the distance image is strongly affected by the level of SA, although aspheric IOLs perform slightly better than their counterparts with a spherical design. For small pupils, there are no differences between the spherical and aspheric IOLs [80]. 

**Figure 15:**
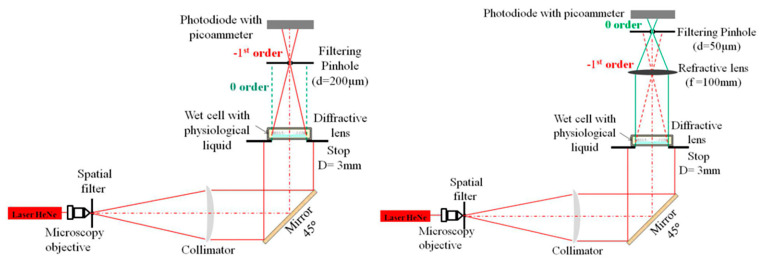
Setup to measure the 1st (**left**) and 0th (**right**) order diffractive efficiency. The 0th order remains collimated behind the diffractive lens and is brought to focus by using an additional convergent lens of 100 mm focal length (**right**), which has a high Strehl ratio (98%). The Strehl ratio S is a suitable figure of merit, defined as the normalized peak intensity of the PSF of the lens: S = I_real_(0,0)/I_ideal_(0,0) = |∫∫e^ikψ(x,y)^ dxdy|^2^ where I_real_(0, 0) and I_ideal_(0, 0) denote the intensities at the center of the real point image and the ideal point spread function (PSF) without aberrations, respectively [19]. Figure reprinted from Ref. [82] under the terms of the Creative Commons Attribution License 4.0.

Slightly more complicated optical bench setups have been proposed to assess/characterize the optical features of advanced model intraocular lenses such as diffractive IOLs. For instance, bifocal diffractive IOLs were obtained by combining two lenses: (1) a carrier lens which determines the power for the far vision and (2) a diffractive profile providing the addition needed to correct the near vision. This approach was widely used in the correction of presbyopia or when cataract surgery was performed [81]. In Figure 15 is shown the optical setup which is useful to measure the 1st and 0th order diffractive efficiency, described in detail in Ref. [82] and briefly reported below. A spatially filtered and collimated HeNe laser (633 nm) beam is used to obtain a smooth and flat wavefront. The optical bench must be vertical (left panel in Figure 15) as the lens floats in its cell. The 0th order remains collimated behind the diffractive lens and is brought to focus by using an additional convergent lens of a 100 mm focal length (right panel in Figure 15), which has a high Strehl ratio (98%). Hence, it is placed at the focus of the −1st order to reduce its contribution in the 0th order efficiency measurement. For each focus, the energy is integrated through a pinhole whose diameter is equal to that of the first ring of the Airy pattern defined by the relation: d = 2.44 λ f_ob_/D (being λ = 633 nm, the wavelength of the excitation beam; D = 3 mm, the diameter of the stop; and f_ob_ the focal length associated with the diffracted order). In the absence of aberration, the first ring of an Airy pattern contains 84% of the total energy; thus, a correction factor must be taken into account to calculate the diffractive efficiency.

**Figure 16:**
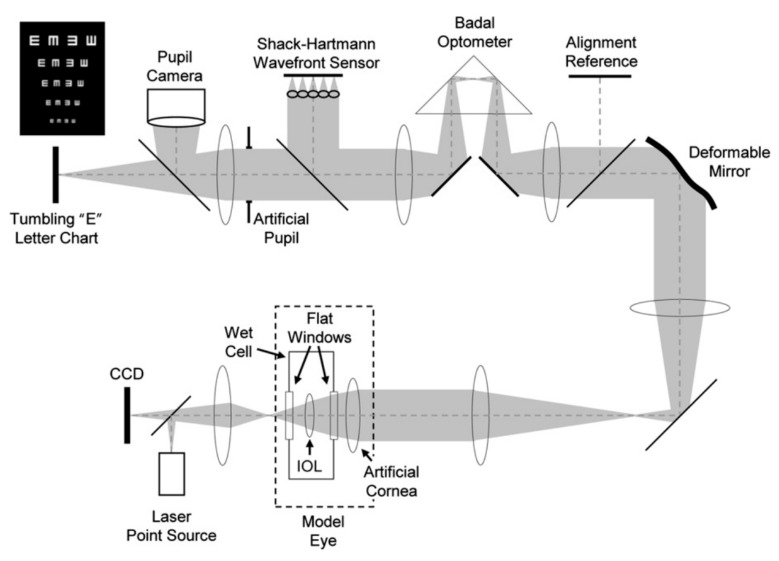
Complete adaptive optics IOL metrology system. Reprinted with permission from Ref. [83]. L. Zheleznyak et al. Impact of corneal aberrations on through-focus image quality of presbyopia-correcting intraocular lenses using an adaptive optics bench system, *Journal of Cataract and Refractive Surgery* 38 (10):1724–1733, 2012, © Wolters Kluwer Health, Inc., Philadelphia, PA, USA.

Generally, the adaptive optics IOL metrology system is characterized by three main sections: a model eye, an imaging arm, and the adaptive optics (see Figure 16, see Ref. [83]). 

In detail: 

**The model eye**: It consists of a wet cell in conjunction with an artificial cornea modelled by an aspheric doublet, as recommended by ISO 11979-2:2014 [78]. The air space between the artificial cornea and the wet cell was set to 4.0 mm; so, the ratio of the entrance pupil diameter to the beam size at the IOL was in accordance with that found in the Gullstrand model eye. The intraocular lens alignment was validated with a pupil camera, where the pupil size is accurately controlled with an artificial pupil located in a relayed pupil plane.

**The imaging arm**: Images of a resolution target consisting of a tumbling letter acuity chart with lines corresponding to 20/40, 20/30, 20/25, 20/20, and 20/15 were captured through the focus. The letter chart was displayed by a computer projector in white light placed at the retinal plane. The model eye’s retinal plane was magnified by a microscope objective onto a 5-megapixel charge-coupled device to improve the pixel sampling.

**The adaptive optics system**: It is incorporated into the optical bench system to induce arbitrary corneal aberration profiles (LOAs and HOAs) onto the pupil plane of the model eye in real time. 

Finally, a large-stroke deformable mirror and a custom-made Shack–Hartmann wavefront sensor was used to verify the aberration induction of the deformable mirror.

We outline that together with the optical design of lenses with the proper shape and the optimized wavefront sensors, the physical properties of the lens-based materials must be optimized [84,85]. In fact, IOL material compositions, their design, and the application of polymer coatings cause significant changes in WF aberrations. Then, suitable optical materials must be adopted to make the polymeric IOL/contact lenses. Among them, the most adopted are: polymethyl-methacrylate (PMMA), hydroxy-ethylmethacrylate (HEMA), silicone, hydrophilic acrylic, hydrophobic acrylic and hydrophilic–hydrophobic copolymer, and hydrogels, which have a high refractive index and excellent mechanical properties (see Table 3) which are useful in reducing the higher-order aberrations [86,87,88,89].

## 5. Applications

### 5.1. Adaptive Optics

The adaptive optics (AO) setup, first used in astronomy, is composed of a wavefront sensor, a deformable mirror, and a control system, strictly connected in a closed loop [90]. The input wavefront signal is analyzed by the control system, which continuously adjusts the needed correction thanks to the deformable mirror, the surface of which was modified by tunable actuators [91]. Babcock introduced the idea of adaptive optics for the first time in 1953, with the aim of compensating astronomical observations [92]. Subsequently, Smirnov proposed to apply the same idea to compensate for ocular aberrations [93].

Currently, the assets provided by adaptive optics are adopted for vision science. The researchers focused on retinal imaging and on testing visual function [94,95]. 

**Figure 17:**
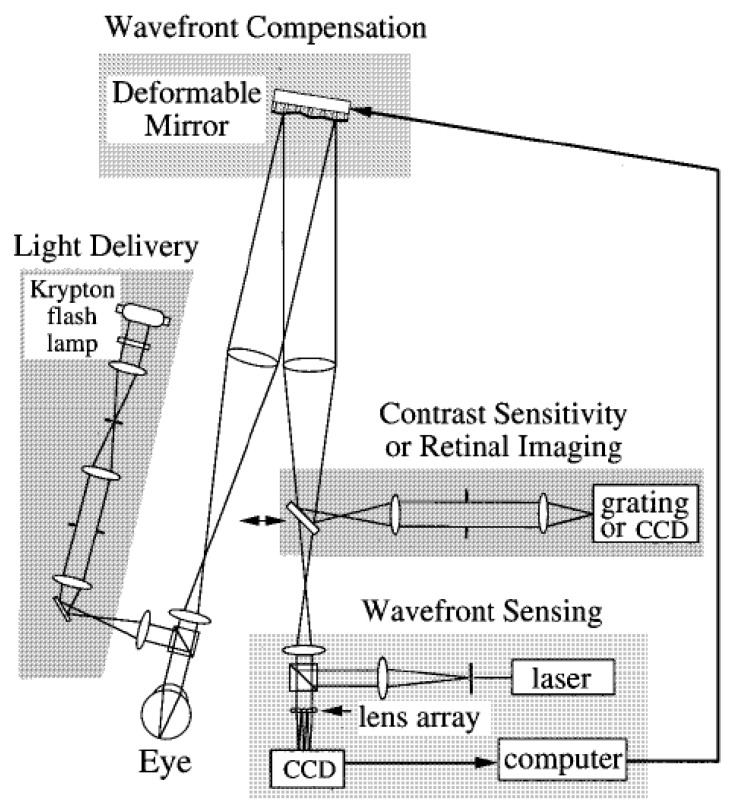
The adaptive system proposed by Williams et al. Figure reprinted with permission from Ref. [95] © The Optical Society.

In 1989, Dreher et al. presented a first adaptive ophthalmological optical system based on a deformable mirror conjugated with the human eye to correct astigmatism [96]. A decade later, Williams et al. achieved the reduction in the Zernike aberrations up to the fourth order, with minor wavefront errors for defocus, astigmatism, coma, and spherical aberrations. As shown in Figure 17, two subsystems were embedded for measuring contrast sensitivity and to perform retinal imaging. Successively, they upgraded the system by increasing the number of actuators in order to correct higher-order aberrations [95,97].

Nevertheless, the scientists are not only interested in studying visual function and its errors but also pathological retinal tissue [98]. Over time, AO for retinal imaging was integrated in clinical use, and recently, a resolution to 2 µm was achieved [98]. For example, Roorda et al. [99] successfully incorporated AO in scanning laser ophthalmoscopes (SLOs). 

**Figure 18:**
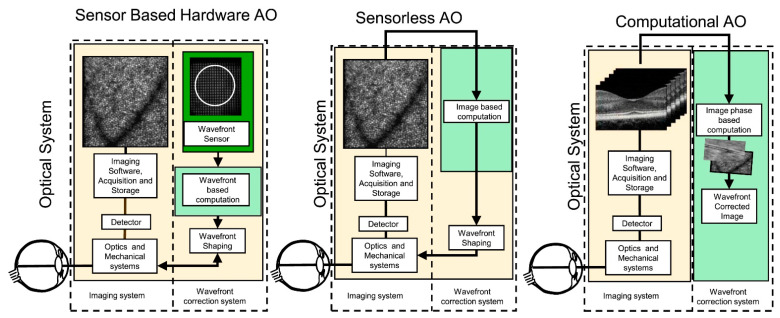
Different categories of AO systems. Reprinted from Ref. [98] Progress in retinal and eye research, 68, S. A. Burns, A. E. Elsner, K. A. Sapoznik, R. L. Warner and T. J. Gast, Adaptive optics imaging of the human retina, pp. 1–30, Copyright (2019) with permission from Elsevier.

The recent technology advancement enables the achievement of AO systems without a wavefront sensor, as in the case of sensorless AO (SAO), and without a wavefront corrector, as in the case of computational AO (CAO). Figure 18 shows the different categories of the AO systems, as extensively described in Ref. [98]. Briefly, in the sensorless setup, the properties of the image are analyzed to adjust the correction needed, whereas, in the computational system, a digital filter is required for the compensation [98].

As will be more evident in the following paragraphs, AO retinal imaging is playing an important role in monitoring both the progression and the treatment of retinal degenerations. AO retinal imaging will continue to be used to investigate diabetes and glaucoma. AO imaging has the potential to improve our understanding and perhaps the monitoring of cerebrovascular and neurodegenerative changes occurring in the retina [100]. Finally, the ability to routinely image cones, rods, and retinal pigment epithelium (RPE) cells will be an important factor in evaluating progression in macular degeneration as well as the impact of therapeutic interventions. 

**Figure 19:**
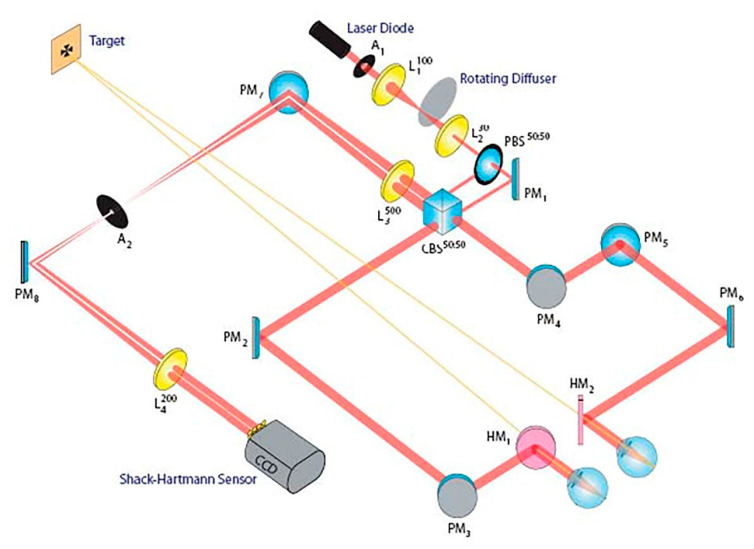
Binocular Shack–Hartmann wavefront sensor. A: aperture, L: lens (superscript represents focal length of the lens in mm), CBS: cube beamsplitter, PBS: pellicle beamsplitter, PM: plane mirror, HM: hot mirror. Figure reused from Ref. [102] under the terms of the OSA Open Access Publishing Agreement, © 2008 Optical Society of America.

The experimental setups previously discussed are designed to study aberrations in monocular vision. Nevertheless, this approach lacks the possibility to study the interaction provided by binocular vision. For this reason, many efforts have been made to develop simultaneous binocular AO systems. In 2009, Fernández et al. proposed a visual simulator that was able to manipulate the aberrations independently in each eye [101]. Their system does not need double components, such as a wavefront sensor and a wavefront corrector, to carry out the simultaneous measurements. In this case, liquid crystal on silicon (LCOS) is used as a wavefront corrector, which modulates the correction needed by modifying the refractive index of the liquid crystal. Instead, binocular infrared optometers allow simultaneous measurement of steady-state accommodation in both eyes, suggesting a significant correlation between the defocus term in the right and left eyes of the same subject. Chin et al. [102], using a binocular Shack–Hartmann wavefront sensor, have measured the ocular wavefront aberrations concurrently in both eyes of six subjects at a sampling rate of 20.5 Hz. More details about the experimental setup, shown in Figure 19, are reported in Ref. [102]. The data analysis procedure follows these main steps: (a) wavefront reconstruction; (b) removal of blink artefacts; and (c) coherence function analysis. So, a dynamic correlation between the ocular wavefront aberrations of two eyes with a binocular Shack–Hartmann wavefront sensor was obtained. Specifically, coherence function analysis shows that the interocular correlation between the aberrations depends on the subject, the Zernike mode, and the frequency and that phase consistency dominates the coherence values.

### 5.2. Clinical Applications of Intraocular Lens Design

#### 5.2.1. Intraocular Lens Design for Wavefront-Shaping Extended Range-of-Vision

**Figure 20:**
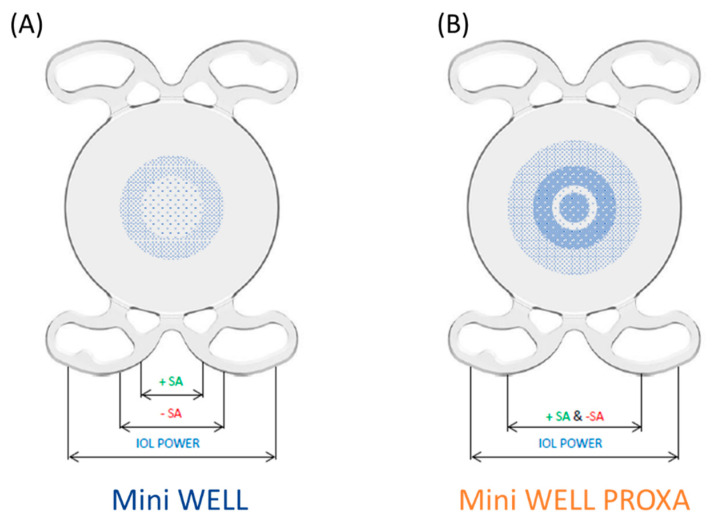
Optical design of (**A**) Mini WELL and (**B**) Mini WELL PROXA. (Courtesy of SIFI SpA).

Nowadays, wavefront technology is strictly connected to the development of new cutting-edge models of intraocular lenses (IOLs). The appropriate use of induced aberrations in extended-depth-of-focus (EDOF) IOLs showed the advantage of enabling the near vision and providing spectacle independence. An example is the MINI WELL (SIFI SpA, Catania, Italy), a non-diffractive EDOF IOL, where positive and negative aberrations are induced in the first two concentric sections, whereas the last ring is monofocal (see Figure 20A) [103,104]. This specific design enables the extension of the depth of focus and the obtaining of a continuous focus range. A good quality of vision is provided between 4 m and 50 cm [104]. Starting from this innovative application, an optical system, denominated WELL Fusion, was developed to fully correct presbyopia. A second intraocular lens, called Mini WELL PROXA, was designed to work synergically with Mini WELL and to extend vision up to 33 cm [105]. The optical design of Mini WELL PROXA entails four annular zones where alternatively positive and negative aberrations are introduced, as well as an external monofocal ring (see Figure 20B) [105]. The binocular system WELL Fusion involves the combined implantation of the two IOLs described above, and it exploits a patented wavefront-engineered technology to provide a good and continuous quality of vision between 4 m and 33 cm [105].

Over the past decade, the interest in EDOF IOLs based on wavefront technology increased and other devices were developed [106,107]

**Figure 21:**
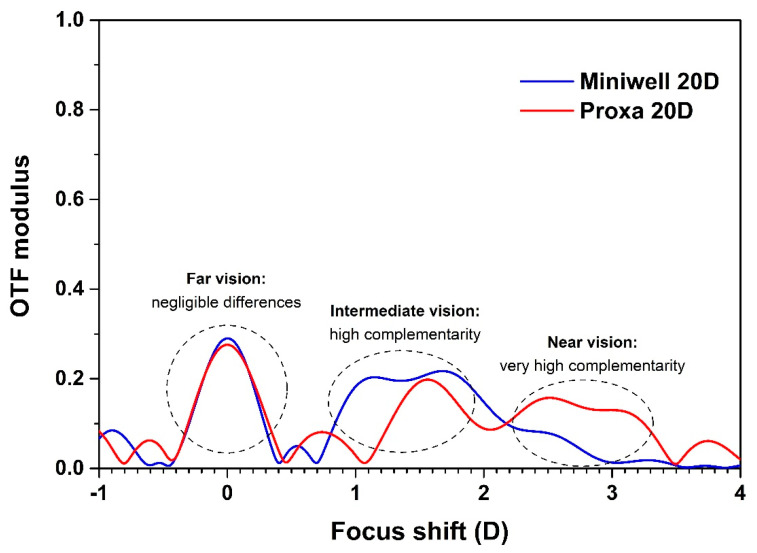
Theoretical Through-Focus Modulation Transfer Function (TF-MTF) curves of Mini WELL and Mini WELL PROXA with a 3 mm aperture and spatial frequency of 50 lp/mm. (Courtesy of SIFI SpA).

Figure 21 reports the theoretical Through-Focus Modulation Transfer Function (TF-MTF) curves for Mini WELL and Mini WELL PROXA [105]. OpticStudio software (Zemax, LLC, Kirkland, WA, USA) was used to simulate the behaviour of both lenses at a spatial frequency equal to 50 lp/mm by considering an Arizona model eye and a 3 mm aperture. As can be seen from the picture, the modulation transfer function is quite similar in the far vision region for both lenses, whereas it provides a complementary response in the intermediate and near vision. As matter of fact, its optics was designed to work jointly and to reach a full presbyopia correction by closing the gap in the near vision up to 33 cm.

The optical quality of MINI WELL was tested and compared with other IOLs on the market. Domínguez-Vicent et al. compared MINI WELL with TECNIS Symfony (Johnson & Johnson Surgical Vision Inc., Santa Ana, CA, USA) in terms of optical quality, such as modulation transfer function (MTF) and through-focus MTF (TF-MTF). Both IOLs provide an extended depth of focus but with different optical designs: TECNIS Symfony exploits an achromatic diffractive platform, whereas MINI WELL introduces spherical aberrations on a non-diffractive surface [108]. The study carried out by Domínguez-Vicent et al. demonstrated that MINI WELL is more defocus-tolerant for intermediate and near distances than TECNIS Symfony, in both photopic and scotopic conditions. This experimental result is consistent with the clinical outcomes, as reported by Nowik et al. in their retrospective observational study [109]. Nowik et al. compared MINI WELL with TECNIS Symfony from the clinical point of view; they found that MINI WELL provides a larger range of depth of focus than TECNIS Symfony, and the difference was statistically significant. Moreover, MINI WELL recorded a lower incidence of dysphotopsia thanks to its non-diffractive optics and a higher percentage of spectacle independence at both close and intermediate distances. 

MINI WELL and Tecnis Symfony were compared by Camps et al. in terms of an “in vitro” aberrometric profile [76]. Camps et al. used an experimental setup, including a Shack–Hartmann wavefront sensor, to obtain Zernike polynomials from the third to the sixth orders. As expected, MINI WELL generated positive and negative spherical aberrations. Camps et al. found that TECNIS Symfony generated some negative spherical aberrations (−0.12 µm) to compensate for the positive primary spherical aberration which is normally present in the cornea.

#### 5.2.2. Refractive Surgery

**Figure 22:**
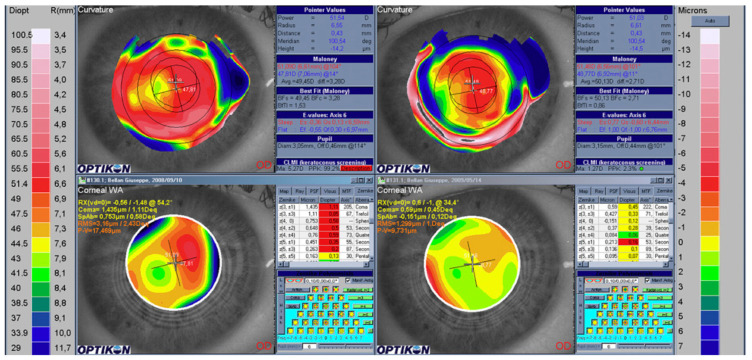
Maps of a post-RK eye. Top left: curvature map before simultaneous PRK–PTK. Top right: curvature map after simultaneous photorefractive keratectomy and phototherapeutic keratectomy (PRK–PTK). Bottom left: corneal wave aberration map before simultaneous PRK–PTK. Bottom right: corneal wave aberration map after simultaneous PRK–PTK. This figure was published in Ref. [112] *Journal of Cataract & Refractive Surgery*, M. Camellin and S.A. Mosquera, Simultaneous aspheric wavefront-guided transepithelial photorefractive keratectomy and phototherapeutic keratectomy to correct aberrations and refractive errors after corneal surgery, 1173–1180, Copyright Elsevier (2010).

Refractive surgery exploits laser ablation to modify the shape of the cornea and consequentially the provided refraction. In clinical practice, refractive surgery is a routine treatment aimed at correcting vision impairment, such as myopia, hyperopia, or astigmatism. Different surgery techniques can be used to obtain the compensation for the refractive errors. Nowadays, laser in situ keratomileusis (LASIK) and photorefractive keratectomy (PRK) are among the most widely used treatments [110]. Unfortunately, traditional refractive surgery could increase higher-order aberrations, especially spherical aberrations [15]. Wavefront-guided refractive surgery avoids the occurrence of this side effect thanks to a previous wavefront analysis. The intended ablation pattern is customized and based on the aberration map of the patient’s eye. A limitation of this technology is the precise alignment with the eye that is critical for a good outcome. Moreover, the success of the surgery highly depends on the healing process; so, the result is unpredictable. Nevertheless, the risk of induced aberrations is lower when compared with traditional refractive surgery, and many studies reported an improved contrast sensitivity and a reduction in halos and glare [23,111]. Figure 22 shows a real case of a patient who underwent a wavefront-guided PRK treatment [112]. The comparison between the preoperative and postoperative topography, together with the statistical analysis, demonstrated the significant decrease in aberrations. 

The introduction of customized refractive surgery was possible thanks to the development of specific technology, taking into account the main limitations due to the dimension of the laser beam and thus the precise spot placement. Small irregularities in the cornea are generally treated using the flying-spot technology characterized by smaller beams (0.5 to 1.0 mm), allowing better and more accurate results in custom ablations to correct irregular astigmatism. However, spot sizes less than 1 mm are required to adequately correct up to the fourth order terms [113]. In some cases, devices with a variable spot size (e.g., VISX S4; Abbott Medical Optics Inc., Santa Ana, CA, USA) are adopted, as well as a device allowing the overlapping of the spots to obtain a smooth surface. In this case, high-speed eye-tracking systems are implemented because of the smaller spot size and also because of the risk of individual pulse decentration and misplacement compared to broad-beam lasers. Centration needs to be accurate as minimal misalignment can induce a completely different aberration pattern. In addition, the scanning-spot frequency must not exceed the rate followed by the tracking system. Finally, it is worth mentioning that other treatments take into consideration the impact of aberrations in refractive surgery, such as the wavefront-optimized profile and the custom Q-factor profile.

The wavefront-optimized profile is based on an aspheric profile designed by Mrochen et al. [114] in order to compensate the aberrations induced by conventional refractive surgery. In fact, the loss in ablation efficacy, due to the angle of incidence of the excimer laser pulses in the midperiphery, can lead to a decrease in the intended ablation depth and, in turn, an increase in spherical aberration [113].

The custom Q-factor profile aims to improve the visual outcome thanks to the manipulation of the corneal asphericity. Manns et al. [115] suggested that a minimum of spherical aberration would be obtained at a target Q-factor of approximately −0.4 [113]. It remains to be seen whether all these treatments are totally beneficial for visual performance. Technology, such as adaptive optics, might be a useful tool to reach a higher level of customization. For example, preoperative patient simulations, with different combinations of aberrations, might help in determining the specific amount and Zernike mode of aberration to target with the treatment [113].

#### 5.2.3. WFS Combined with Ophthalmic Technologies

One of the causes of blindness is the dysfunction of the blood–retinal barrier, typically observed in people affected by diabetic retinopathy [116], whose study requires a high optical resolution (6.5 μm in diameter [117]) to visualize single capillaries and the blood cells which, in the imperfect optics of the mammalian anterior eye, induce aberrations that blur the microscopic retina features. Even if the AO ophthalmoscopy has enabled diffraction-limited imaging of the retina by measuring and correcting for higher- and lower-order aberrations of the eye, the single blood cell imaging still cannot still be easily observed. The movement of the blood cell limits the acquisition which can be made using high-frame rate-cameras [118]. Furthermore, fast cameras require even more light, which could damage the eye tissue. 

**Figure 23:**
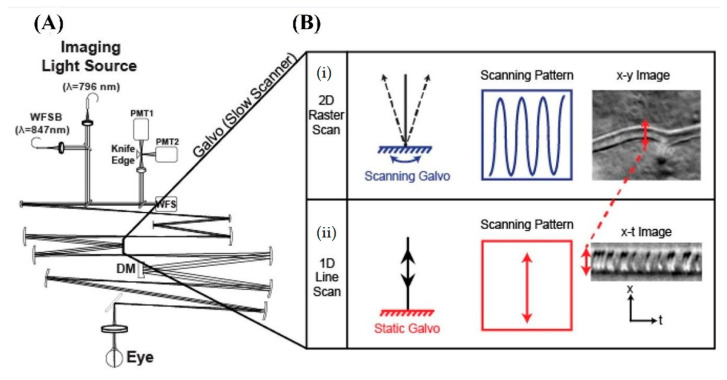
(**A**) Scheme of the scanning imaging system proposed by Guevara-Torres et al., composed of 5 pairs of afocal telescopes that relay coaligned beams for imaging and wavefront sensing. (**B**) The two possible scanning modes: (**i**) 2D raster scan and (**ii**) 1D line scan. See main text for more details. Figure reused from Ref. [119] under the terms of the OSA Open Access Publishing Agreement, © 2016 Optical Society of America.

To overcome this drawback, Guevara-Torres et al. [119] developed a scanning imaging system allowing the collection of 2-dimensional raster images at a rate of 25 frames per second, with 1D fast scanning operating at 15.45 kHz. This setup was composed of five pairs of afocal telescopes that relayed coaligned beams for imaging and wavefront sensing. The 843 nm or 904 nm laser sources were used. In the return path, light is reflected into high-sensitivity photomultiplier tubes (Figure 23) and, in real time, the eye aberrations were measured with a Shack–Hartmann wavefront sensor, corrected with a deformable mirror.

Dynamic and static wavefront aberrations influence retinal OCT image quality across a wide and limited field of view (FOV). Actually, the optical coherence tomography angiography (OCTA) has become an increasingly important tool for diagnosing retinal parafoveal microvasculature and vein occlusion. In particular, the adaptive optics with closed-loop feedback—wherein a wavefront sensor detects, and a deformable mirror compensates, optical aberrations—have been considered as a potential solution [120]. Polans et al. proposed a compact OCTA system integrated with wavefront sensorless adaptive optics (WSAO). The wide-field OCTA system covers a 70° field of view, ultimately allowing the correction of peripheral aberrations within 2 s to a level that was sufficient for the enhanced visualization of microvasculatures and microaneurysms in diabetic patients.

**Figure 24:**
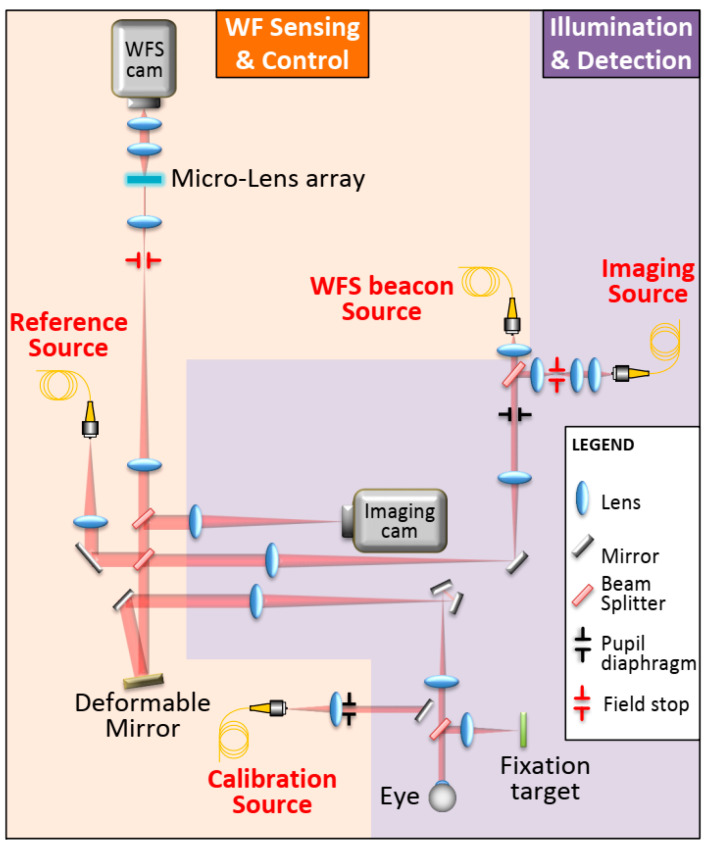
Scheme of AO flood illumination ophthalmoscope. Figure reused from Ref. [124] under the terms of the OSA Open Access Publishing Agreement, © 2019 Optical Society of America.

Recently, some researchers have worked to optimize the image processing approach which is useful for generating retinal perfusion maps adapted to image sequences obtained with AO-corrected ophthalmoscopes [121,122]. However, in the contrast maps some artifacts are present, which implies an uncertainty as to whether a movement observed between two frames is due to physiological reasons or due to scan distortion [123]. Moreover, some other drawbacks should be still overcome, such as a small field of view, an uneven contrast in the capillaries, or a limitation concerning the direction and plane of the vessels whose blood flow can be analyzed. Salas et al. [124] developed a computational approach, relying on a spatio-temporal filtering of the image sequence, which is useful for isolating blood flow from noise in low-contrast sequences. Applying this computational approach, angiography with an adaptive optics flood illumination ophthalmoscope (AO-FIO) using NIR light, in both bright-field and dark-field modalities, has been carried out [124]. Figure 24 reports a scheme of the AO flood illumination ophthalmoscope, arranged in two parts: (1) wavefront (WF) sensing and control and (2) illumination and detection. The first is composed of a reference source (Ref Source), a wavefront sensor (WFS) (microlens array, relay optics, and WFS camera), a WFS beacon source, and a deformable mirror (DM). An additional calibration source can be inserted in place of the eye to calibrate the adaptive optics loop. The illumination and detection subsystem is composed of the retinal imaging camera and the corresponding wide-field imaging source.

As outlined by Piñero et al. [125], the consistency of the refractive measurements is not dependent on the magnitude of the refractive error, with the same precision ability for moderate to high myopia and for hyperopia. In the last few years, teams of researchers have adopted the Visionix VX120 (Luneau Technologies SAS, Pont-de-l’Arche, France), a multi-diagnostic platform providing consistent measurements of refraction, keratometry, central corneal thickness (CCT), and iridocorneal angle (IA) in normal healthy eyes. This non-invasive and high level of intra- and inter-session repeatability, multi-diagnostic platform combines refraction (Hartmann–Shack-based autorefractometer), simulated keratometry (based on Placido disk videokeratography), non-invasive stationary Scheimpflug-based pachymetry, and Hartmann–Shack wavefront aberrometry (see Ref. [126]). So, a complete exam of the anterior segment of the eye (cataracts, refractive error screening, glaucoma screening and monitoring, adaptation of rigid and scleral contact lenses, keratoconus stage classification and monitoring, and complete readings for keratometry and night vision) could be made. 

Recently, François Hénault et al. [127] proposed a crossed-sine wavefront sensor which is useful for simultaneously achieving a high spatial resolution at the pupil of the tested optics and absolute measurement accuracy comparable to that attained by laser interferometers. This is obtained using a linear gradient transmission filter (GTF), located at the image plane of the tested optical system, the mini-lens array, and a detector array, thus allowing the acquisition of four pupil images simultaneously. The authors also carried out numerical simulations in order to assess the performance of the crossed-sine WFS in terms of measurement accuracy. The accuracy of the crossed-sine WFS is better than λ/100 RMS, which is significantly higher than that offered by commercial WFS (typically λ/25 RMS). Furthermore, the crossed-sine WFS offers the advantage of being quasi-achromatic and able to work on slightly extended illumination objects, thus allowing a vast choice of natural or artificial sources.

**Figure 25:**
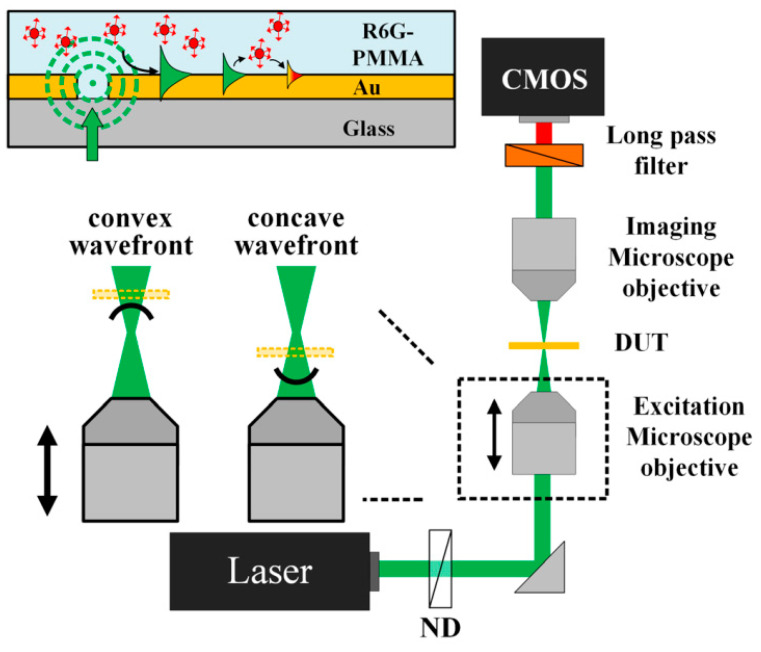
The confocal configuration used to measure the shift in the focal spot. ND is a Neutral Density filter and DUT is the Device Under Test. Figure reprinted from Ref. [128] Charles Pelzman and Sang-Yeon Cho, “Wavefront detection using curved nanoscale apertures”, *Applied Physics Letters* 114, 183103 (2019), with the permission of AIP Publishing.

For future technological applications, we recall the paper of Pelzman et al. [128]. Generally, a multi-lens setup and several images are necessary to measure the wavefront using the sensors previously described. For example, the pyramid sensor requires a lenslet array as well as a mechanical vibrating crystal, and the Shack–Hartmann sensor uses an array of micrometer-scale lenslets to convert the wavefront information of an incoming beam into a two-dimensional intensity map made out of focused spots. Thus, an optimization of the optical alignment of the lenslet array and a focal plane array are fundamental steps to carry out. On the other hand, an ultrahigh spatial resolution is the peak demand today in wavefront detection. It is well known that the excited SP waves in subwavelength structures still carry the wavefront information of the incident wave, according to Huygens–Fresnel principle [129]. Starting from this principle and using a concentric-ring-based aperture array fabricated in an Au film, Pelzman et al. have developed a device showing wavefront-dependent focusing of the surface plasmon (SP) waves. In addition, the demonstrated approach does not require complicated 3D integration or optical alignment, and thus, it has great potential for revolutionizing the existing wavefront sensing technologies. Figure 25 shows the confocal configuration used to measure the shift in the focal spot. The shape of the incident wavefront is easily controlled through defocusing the excitation beam while maintaining the imaging objective in focus. 

Specifically, by intentionally defocusing the excitation beam, the shape of the incident wavefront was converted from convex to concave. The inset of Figure 25 shows how the SP waves excited (by a diode-pumped 532-nm laser) on the surface interact with the fluorescent dye molecule embedded in the PMMA layer. Then, the emitted fluorescence signal from the interaction of the suspended R6G molecules with the SP waves was collected through the imaging microscope objective. A long-pass optical filter with an edge wavelength of 550 nm was used to block the optical signal from the 532 nm excitation line. Some details are reported in Ref. [128].

Recently, an innovative approach was based on the use of artificial metamaterials, known as metasurfaces, which can impart a phase shift on transmitted or reflected light, allowing for unconventional beam shaping over subwavelength distances [130,131]. Recalling once again the principle of Huygens–Fresnel, a physical implementation of Huygens’ fictitious sources can be realized by engineering crossed electric and magnetic dipoles and thus providing full transmission with the arbitrary 2π phase and, in turn, allowing extreme control and manipulation of light [132,133,134,135]. In detail, the atomic array, producing diffraction-limited focusing of light with very short wavelength-scale focal lengths, was simulated using the coherently superposing induced electric and magnetic dipoles. In this way, a quantum nanophotonic Huygens surface of atoms was engineered obtaining a full 2π phase control over the transmission, with close to zero reflection. In view of the diffraction-limited focusing, atomic arrays offer advantages over plasmonic or dielectric platforms (i.e., the absence of absorptive loss and fabrication inhomogeneities and a great flexibility to operate at the quantum limit) [136].

**Figure 26:**
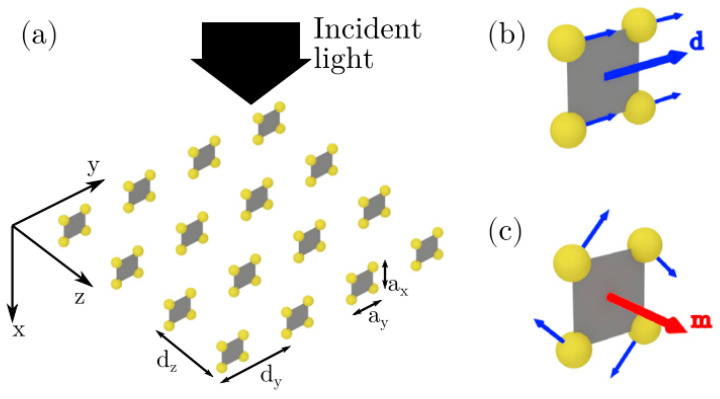
(**a**) Two-dimensional square lattice constituting an atomic Huygens surface with four atoms per unit cell. (**b**) An electric dipole moment, d (given by the vectorial sum of the individual dipole moments identified by the ticker blue arrows), is generated by the uniform polarization in the unit cell. (**c**) A magnetic dipole moment, m, is generated by the azimuthal polarization. Figure reused from Ref. [136] under the terms of the Creative Commons Attribution 4.0 International License.

A representative atomic Huygens surface with strong magnetic response at optical frequencies is shown in Figure 26, as reported in Ref. [136]. The atomic array consists of a 2D square lattice in the yz plane. Each site consists of a square unit cell of four atoms, forming an atomic bilayer. In Figure 26b,c are also reported a scheme indicating how a uniform polarization on each atom leads to an effective electric dipole moment d from the unit cell, while an azimuthal polarization leads to a net zero electric dipole moment, and to a perpendicular magnetic dipole moment m.

## 6. Wavefront Sensing Technology to Empower Clinical Ophthalmic Surgery Application of Multifocal IOLs: Future Developments

Wavefront technology has the potential to help us truly assess and understand how and what the patient really sees. With this more comprehensive understanding of the patient’s aberrations comes an increased capacity and responsibility to correct them. Furthermore, wavefront sensing technology empowers the surgeon to ensure that the IOL implanted is the one that will achieve the refractive outcomes which are unique to each patient’s visual needs. As cataract surgery has evolved into lens-based refractive surgery, expectations for refractive outcomes continue to increase with a wide variety of options to correct refractive error. 

As already mentioned in the previous sections, in the clinical setting wavefront systems are generally used in combination with corneal topographers to evaluate the aberrations of the patient’s eye in the preparation for LASIK treatment or the implantation of IOLs in the pre-operative and post-operative follow-up phases. Today, new keratorefractive techniques such as small incision lenticule extraction (SMILE) avoid corneal flap creation and use a single laser device, while advances in surface ablation techniques have seen a resurgence in popularity. Presbyopic treatment options have also expanded to include new ablation profiles, intracorneal implants, and phakic intraocular implants. For all these approaches, a pre-operative evaluation of refractive patients is strongly necessary. Recently, this evaluation has been carried out by using machine learning and artificial intelligence [137], in which multiple diagnostic tools receive information about the eye and guide the surgeon regarding the lens or the best corneal refractive surgery method to perform on a specific patient to adequately correct the refractive error, improving the quality of the retinal image to beyond normal levels. 

**Figure 27:**
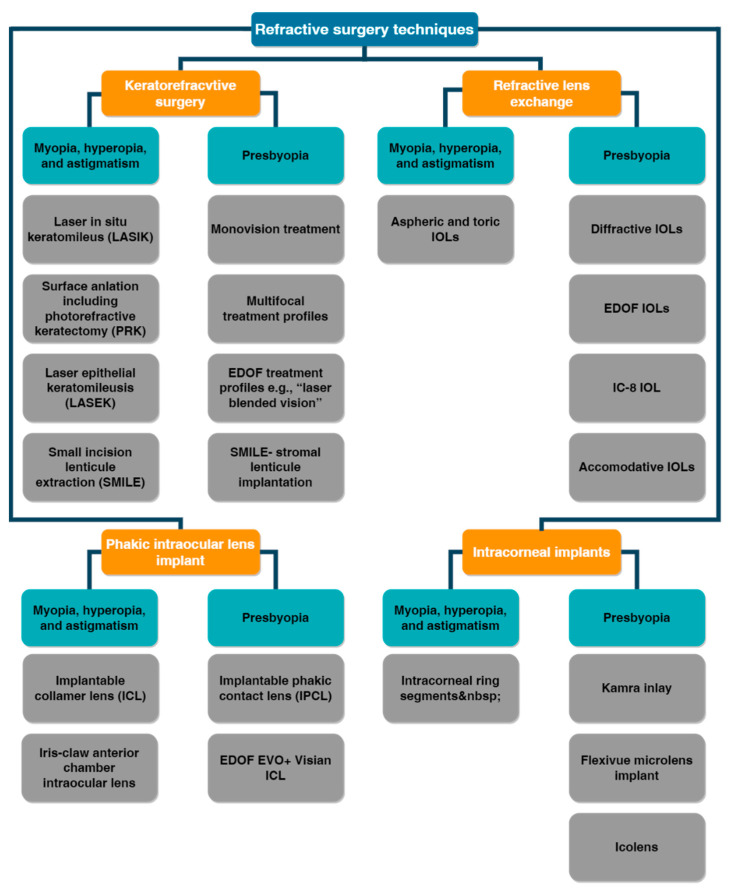
Scheme of refractive surgery techniques. For more details, see Ref. [137].

Figure 27 shows a summary of the most widely used refractive surgery techniques. For example, conventional LASIK is useful to correct lower-order aberrations, such as defocus and astigmatism, but it is not adequate for patients with other distortions, such as halos, glare, and impaired night vision. This is because, with conventional LASIK, we are unable to see the true complexity and the interrelationship of the aberrations. In fact, we can see different aberrations independently, but we have no complete map of their relationship with one another. A further complicating factor is that the amount of higher-order aberrations the population experiences is not at all related to the level of myopia. In other words, patients with –1 D can have just as many higher-order aberrations as those with –8 D. This means that refractive surgery that addresses only the sphere and the cylinder may not improve a patient’s overall vision. On the other hand, to date, custom ablation allows us to avoid increasing spherical aberration, thereby significantly improving halos at night. Studies have found that patients treated with custom ablation experience improvements in glare, halo, night driving, blurred vision, and fluctuation of vision.

Recently, Alcon launched the Optiwave Refractive Analysis (ORA) system (Alcon Laboratories, Inc., Fort Worth, TX, USA), which optimizes intraoperative wavefront data to calculate IOL power and helps with IOL selection. It exploits Talbot Moiré interferometry to provide accurate real-time information during surgery [138]. It also includes analytical tools to evaluate results compared to an aggregate global database [139].

Moreover, although the expansion of the optometric scope of care may have drifted the profession away from the traditional roots of physiological optics and towards the treating and managing of ocular disease, non-surgical wavefront correction provides evidence that once again refractive error is an appealing and central part. Most importantly, patients will benefit from better visual quality with the least invasive solution. In fact, with wavefront analysis we can really see the whole problem and treat it as such and begin to understand that not everyone’s visual map is the same. 

Here, we outline that wavefront-guided devices do not stop with custom ablation. Wavefront devices have come a long way since the original bulky prototypes first used, and now researchers are experimenting with numerous exciting prospects [140,141,142]. Now that some of the devices are so small, the possibilities are virtually limitless. One idea that is currently in development is wavefront-guided contact lenses, which could be customized to the individual’s eye using digital information. Another possibility is to adjust IOLs digitally inside the eye with a wavefront device.

Together with the wavefront technologies, particular attention should be paid to intraocular lenses (IOLs). As reported in Section 5.2, IOLs represent the most advanced solution for cataract refractive surgery. The most advanced IOLs for this purpose are the EDOF (extended depth of focus) lenses that present an optical plate with a continuous series of focuses to ensure a continuum correction from far to near in the case of presbyopia and, in case of astigmatism, to provide compensation for the corneal abnormal curvature. 

This field is still the subject of analysis and prototyping. As the IOL trend is oriented towards lenses of increasing complexity, it is necessary to have wavefront analysis instrumentation that is, in turn, able to follow the complexity of the lens to allow its validation, compliance with the optical design, quality control, and consistency with production batches. This need forces the development trend towards the wavefront in AO technology.

## 7. Conclusions

The selection of the most adequate AO wavefront sensing detectors is essential to analyze the optical retinal imaging modalities and the IOL/contact lenses performance. Nowadays, to compensate for the light aberrations, adaptive optics (AO), a technology initially developed in astronomy, is largely utilized. In this review, we first reported on an overview of the main wavefront sensors planned to be a part of the many instruments that are currently under development for AO applications, and we described their advantages and limitations. In the second part of this review, we outlined selected applications of the IOL and AO systems and the issues that have to be solved to approach the high performance of the optical systems as well as the high degree of process control that is required in AO applications. Finally, the directions for further investigations are reported with regard to the potential of the new materials, whose physical properties are particularly interesting for creating new designs and optimizing the performance of IOLs and AO systems. To this end, further studies closely combining the features of wavefront science with the application demands of the various functional materials are still necessary.

## Figures and Tables

**Table 3:** IOL materials and their properties.

IOL Material	Sphericity Status	Hygroscopy (%)	Glass Transition Temperature (°C)	Refractive Index (*n*)
Collamer	Negative	40	40	1.44
Hydrophobic acrylic	Spherical	0.1–0.5	16–55	1.47–1.56
Hydrophilic acrylic	Neutral	18–38	10–20	1.40–1.43
PEG-PEA/HEMA/Styrene copolymer	Neutral	4–5	28	1.54
PMMA	Negative	0.4–0.8	105–113	1.49
Silicone	Neutral	0.38	−90–−120	1.43

## Data Availability

Data are available upon reasonable request by the corresponding author.

## References

[1] Kugler L.J., Wang M.X. (2010). Lasers in Refractive Surgery: History, Present, and Future. Appl. Opt..

[2] Kligman B.E., Baartman B.J., Dupps W.J. (2016). Errors in Treatment of Lower-Order Aberrations and Induction of Higher-Order Aberrations in Laser Refractive Surgery. Int. Ophthalmol. Clin..

[3] Maeda N. (2009). Clinical Applications of Wavefront Aberrometry—A Review. Clin. Exp. Ophthalmol..

[4] Charman W.N. (2005). Wavefront Technology: Past, Present and Future. Contactlens Anterior Eye.

[5] Briguglio R.A., Agapito G., del Vecchio C., Pinna E., Xompero M., Arcidiacono C., Terreri A., Pedichini F., Sodnik Z., Cugny B., Karafolas N. (2021). Demonstrating the Sub-Nanometer Sensitivity of a Pyramid WaveFrontSensor for Active Space Telescopes. Proceedings of the International Conference on Space Optics—ICSO 2020.

[6] Perrin M.D., Acton D.S., Lajoie C.-P., Knight J.S., Lallo M.D., Allen M., Baggett W., Barker E., Comeau T., Coppock E., MacEwen H.A., Fazio G.G., Lystrup M., Batalha N., Siegler N., Tong E.C. (2016). Preparing for JWST Wavefront Sensing and Control Operations. Proc. SPIE 9904, Space Telescopes and Instrumentation 2016: Optical, Infrared, and Millimeter Wave.

[7] Maeda N. (2001). Wavefront Technology in Ophthalmology. Curr. Opin. Ophthalmol..

[8] Ryan D.S., Sia R.K., Rabin J., Rivers B.A., Stutzman R.D., Pasternak J.F., Eaddy J.B., Logan L.A., Bower K.S. (2018). Contrast Sensitivity After Wavefront-Guided and Wavefront-Optimized PRK and LASIK for Myopia and Myopic Astigmatism. J. Refract. Surg..

[9] Piñero D.P., Soto-Negro R., Ruiz-Fortes P., Pérez-Cambrodí R.J., Fukumitsu H. (2019). Analysis of Intrasession Repeatability of Ocular Aberrometric Measurements and Validation of Keratometry Provided by a New Integrated System in Mild to Moderate Keratoconus. Cornea.

[10] Hampson K.M. (2008). Adaptive Optics and Vision. J. Mod. Opt..

[11] Yoon G. (2005). Wavefront Sensing and Diagnostic Uses. Adaptive Optics for Vision Science.

[12] Bille J.F., Harner C.F., Lösel F., Bille J.F., Harner C.F.H., Loesel F.F. (2004). Aberration-Free Refractive Surgery: New Frontiers in Vision.

[13] Lakshminarayanan V., Fleck A. (2011). Zernike Polynomials: A Guide. J. Mod. Opt..

[14] Applegate R.A. (2004). Glenn Fry Award Lecture 2002: Wavefront Sensing, Ideal Corrections, and Visual Performance. Optom. Vis. Sci..

[15] Lombardo M., Lombardo G. (2009). New Methods and Techniques for Sensing the Wave Aberrations of Human Eyes. Clin. Exp. Optom..

[16] He J.C., Burns S.A., Marcos S. (2000). Monochromatic Aberrations in the Accommodated Human Eye. Vis. Res..

[17] Chen L., Singer B., Guirao A., Porter J., Williams D.R. (2005). Image Metrics for Predicting Subjective Image Quality. Optom. Vis. Sci..

[18] Harvey J.E., Ftaclas C. (1995). Diffraction Effects of Telescope Secondary Mirror Spiders on Various Image-Quality Criteria. Appl. Opt..

[19] Ottevaere H., Thienpont H. (2005). Optical microlenses. Encyclopedia of Modern Optics.

[20] Lawless M.A., Hodge C. (2005). Wavefront’s Role in Corneal Refractive Surgery. Clin. Exp. Ophthalmol..

[21] Wang K., Xu K. (2021). A Review on Wavefront Reconstruction Methods. Proceedings of the 2021 4th International Conference on Information Systems and Computer Aided Education.

[22] Li Z., Li X., Gao Z., Jia Q. (2021). Review of Wavefront Sensing Technology in Adaptive Optics Based on Deep Learning. High Power Laser Part. Beams.

[23] Mello G.R., Rocha K.M., Santhiago M.R., Smadja D., Krueger R.R. (2012). Applications of Wavefront Technology. J. Cataract Refract. Surg..

[24] McKay G.N., Mahmood F., Durr N.J. (2019). Large Dynamic Range Autorefraction with a Low-Cost Diffuser Wavefront Sensor. Biomed. Opt. Express.

[25] Greivenkamp J.E., Smith D.G., Gappinger R.O., Williby G.A., Iwata K. (2001). Optical Testing Using Shack-Hartmann Wavefront Sensors. Proceedings of the Optical Engineering for Sensing and Nanotechnology (ICOSN ’01).

[26] Haffert S.Y. (2016). Generalised Optical Differentiation Wavefront Sensor: A Sensitive High Dynamic Range Wavefront Sensor. Opt. Express.

[27] Iglesias I., Ragazzoni R., Julien Y., Artal P. (2002). Extended Source Pyramid Wave-Front Sensor for the Human Eye. Opt. Express.

[28] Swain B.R., Dorrer C., Qiao J. (2019). High-Performance Optical Differentiation Wavefront Sensing towards Freeform Metrology. Opt. Express.

[29] Oti J.E., Canales V.F., Cagigal M.P. (2005). Improvements on the Optical Differentiation Wavefront Sensor. Mon. Not. R. Astron. Soc..

[30] Berto P., Rigneault H., Guillon M. (2017). Wavefront Sensing with a Thin Diffuser. Opt. Lett..

[31] Xue S., Chen S., Fan Z., Zhai D. (2018). Adaptive Wavefront Interferometry for Unknown Free-Form Surfaces. Opt. Express.

[32] Mrochen M., Kaemmerer M., Mierdel P., Krinke H.-E., Seiler T. (2000). Principles of Tscherning Aberrometry. J. Refract. Surg..

[33] Ragazzoni R., Farinato J. (1999). Sensitivity of a Pyramidic Wave Front Sensor in Closed Loop Adaptive Optics. Astron. Astrophys..

[34] Chanteloup J.-C. (2005). Multiple-Wave Lateral Shearing Interferometry for Wave-Front Sensing. Appl. Opt..

[35] Wiley W.F., Bafna S. (2011). Intra-Operative Aberrometry Guided Cataract Surgery. Int. Ophthalmol. Clin..

[36] Solomon K.D., Fernandez De Castro L.E., Sandoval H.P., Vroman D.T. (2004). Comparison of Wavefront Sensing Devices. Ophthalmol. Clin. N. Am..

[37] Hartmann J. (1900). Bemerkungen Über Den Bau Und Die Justirung von Spektrographen. Zt. Instrum..

[38] Shack R.V., Platt B.C. (1971). Production and Use of a Lenticular Hartmann Screen. J. Opt. Soc. Am..

[39] Rasouli S., Dashti M., Ramaprakash A.N. (2010). An Adjustable, High Sensitivity, Wide Dynamic Range Two Channel Wave-Front Sensor Based on Moiré Deflectometry. Opt. Express.

[40] Platt B.C., Shack R. (2001). History and Principles of Shack-Hartmann Wavefront Sensing. J. Refract. Surg..

[41] Rousset G., Roddier F. (1999). Wave-Front Sensors. Adaptive Optics in Astronomy.

[42] Shinto H., Saita Y., Nomura T. (2016). Shack–Hartmann Wavefront Sensor with Large Dynamic Range by Adaptive Spot Search Method. Appl. Opt..

[43] Akondi V., Dubra A. (2021). Shack-Hartmann Wavefront Sensor Optical Dynamic Range. Opt. Express.

[44] Ehrenfest P. (1927). Notes on the Approximate Validity of Quantum Mechanics. Z. Phys..

[45] Cook R.J. (1975). Beam Wander in a Turbulent Medium: An Application of Ehrenfest’s Theorem. J. Opt. Soc. Am..

[46] Bará S. (2007). Characteristic Functions of Hartmann-Shack Wavefront Sensors and Laser-Ray-Tracing Aberrometers. J. Opt. Soc. Am. A.

[47] Thibos L.N. (2000). Principles of Hartmann-Shack Aberrometry. Vision Science and its Applications.

[48] Moreno-Barriuso E., Marcos S., Navarro R., Burns S.A. (2001). Comparing Laser Ray Tracing, the Spatially Resolved Refractometer, and the Hartmann-Shack Sensor to Measure the Ocular Wave Aberration. Optom. Vis. Sci..

[49] Wu Y., Sharma M.K., Veeraraghavan A. (2019). WISH: Wavefront Imaging Sensor with High Resolution. Light Sci. Appl..

[50] Burvall A., Daly E., Chamot S.R., Dainty C. (2006). Linearity of the Pyramid Wavefront Sensor. Opt. Express.

[51] Ojeda-Castaeda J. (1992). Foucault, Wire, and Phase Modulation Tests. Optical Shop Testing.

[52] Wang H., Liu C., He X., Pan X., Zhou S., Wu R., Zhu J. (2014). Wavefront Measurement Techniques Used in High Power Lasers. High Power Laser Sci. Eng..

[53] Oti J., Canales V., Cagigal M. (2003). Analysis of the Signal-to-Noise Ratio in the Optical Differentiation Wavefront Sensor. Opt. Express.

[54] Oti J.E., Canales V.F., Cagigal M.P., Valle P.J., Jiang W. (2005). Wavefront Sensing by Optical Differentiation. Proceedings of the 5th International Workshop on Adaptive Optics for Industry and Medicine.

[55] Bortz J.C. (1984). Wave-Front Sensing by Optical Phase Differentiation. J. Opt. Soc. Am. A.

[56] Qiao J., Mulhollan Z., Dorrer C. (2016). Optical Differentiation Wavefront Sensing with Binary Pixelated Transmission Filters. Opt. Express.

[57] Sinjab M.M., Cummings A.B. (2018). Introduction to Wavefront Science. Customized Laser Vision Correction.

[58] Shatokhina I., Hutterer V., Ramlau R. (2020). Review on Methods for Wavefront Reconstruction from Pyramid Wavefront Sensor Data. J. Astron. Telesc. Instrum. Syst..

[59] Ragazzoni R. (1996). Pupil Plane Wavefront Sensing with an Oscillating Prism. J. Mod. Opt..

[60] Costa J.B., Ragazzoni R., Ghedina A., Carbillet M., Verinaud C., Feldt M., Esposito S., Puga E., Farinato J., Wizinowich P.L., Bonaccini D. (2003). Is There Need of Any Modulation in the Pyramid Wavefront Sensor?. Proceedings of SPIE Volume 4839, Adaptive Optical System Technologies II.

[61] Díaz-Doutón F., Pujol J., Arjona M., Luque S.O. (2006). Curvature Sensor for Ocular Wavefront Measurement. Opt. Lett..

[62] Torti C., Gruppetta S., Diaz-santana L. (2008). Wavefront Curvature Sensing for the Human Eye. J. Mod. Opt..

[63] Fienup J.R., Thelen B.J., Paxman R.G., Carrara D.A., Bonaccini D., Tyson R.K. (1998). Comparison of Phase Diversity and Curvature Wavefront Sensing. Proceedings of SPIE Volume 3353, Adaptive Optical System Technologies.

[64] Lombaert H., Grady L., Pennec X., Ayache N., Cheriet F. (2014). Spectral Log-Demons: Diffeomorphic Image Registration with Very Large Deformations. Int. J. Comput. Vis..

[65] Gunjala G., Sherwin S., Shanker A., Waller L. (2018). Aberration Recovery by Imaging a Weak Diffuser. Opt. Express.

[66] Baik S.-H., Park S.-K., Kim C.-J., Cha B. (2007). A Center Detection Algorithm for Shack–Hartmann Wavefront Sensor. Opt. Laser Technol..

[67] Shirai T., Barnes T.H., Haskell T.G. (2000). Adaptive Wave-Front Correction by Means of All-Optical Feedback Interferometry. Opt. Lett..

[68] Primot J. (1993). Three-Wave Lateral Shearing Interferometer. Appl. Opt..

[69] Sekine R., Shibuya T., Ukai K., Komatsu S., Hattori M., Mihashi T., Nakazawa N., Hirohara Y. (2006). Measurement of Wavefront Aberration of Human Eye Using Talbot Image of Two-Dimensional Grating. Opt. Rev..

[70] Kim M.-S., Scharf T., Menzel C., Rockstuhl C., Herzig H.P. (2012). Talbot Images of Wavelength-Scale Amplitude Gratings. Opt. Express.

[71] Lombardo M., Serrao S., Devaney N., Parravano M., Lombardo G. (2012). Adaptive Optics Technology for High-Resolution Retinal Imaging. Sensors.

[72] Salama N.H., Patrignani D., de Pasquale L., Sicre E.E. (1999). Wavefront Sensor Using the Talbot Effect. Opt. Laser Technol..

[73] van Heugten A. (2004). Ophthalmic Talbot-Moire Wavefront Sensor. U.S. Patent.

[74] Moreno-Barriuso E., Lloves J.M., Marcos S., Navarro R., Llorente L., Barbero S. (2001). Ocular Aberrations before and after Myopic Corneal Refractive Surgery: LASIK-Induced Changes Measured with Laser Ray Tracing. Investig. Ophthalmol. Vis. Sci..

[75] Tan B., Chen Y.-L., Baker K., Lewis J.W., Swartz T., Jiang Y., Wang M. (2007). Simulation of Realistic Retinoscopic Measurement. Opt. Express.

[76] Camps V.J., Tolosa A., Piñero D.P., de Fez D., Caballero M.T., Miret J.J. (2017). In Vitro Aberrometric Assessment of a Multifocal Intraocular Lens and Two Extended Depth of Focus IOLs. J. Ophthalmol..

[77] Alba-Bueno F., Vega F., Millán M.S. (2011). Design of a Test Bench for Intraocular Lens Optical Characterization. J. Phys. Conf. Ser..

[78] (2014). Ophthalmic Implants—Intraocular Lenses—Part 2: Optical Properties and Test Methods. https://www.iso.org/standard/55682.html.

[79] Son H.S., Labuz G., Khoramnia R., Merz P., Yildirim T.M., Auffarth G.U. (2020). Ray Propagation Imaging and Optical Quality Evaluation of Different Intraocular Lens Models. PLoS ONE.

[80] Vega F., Alba-Bueno F., Millán M.S. (2011). Energy Distribution between Distance and Near Images in Apodized Diffractive Multifocal Intraocular Lenses. Investig. Ophthalmol. Vis. Sci..

[81] Cohen A.L. (1993). Diffractive Bifocal Lens Designs. Optom. Vis. Sci..

[82] Tankam P., Lépine T., Castignoles F., Chavel P. (2012). Optical Metrology for Immersed Diffractive Multifocal Ophthalmic Intracorneal Lenses. J. Eur. Opt. Soc. Rapid Publ..

[83] Zheleznyak L., Kim M.J., MacRae S., Yoon G. (2012). Impact of Corneal Aberrations on Through-Focus Image Quality of Presbyopia-Correcting Intraocular Lenses Using an Adaptive Optics Bench System. J. Cataract Refract. Surg..

[84] Luo C., Wang H., Chen X., Xu J., Yin H., Yao K. (2022). Recent Advances of Intraocular Lens Materials and Surface Modification in Cataract Surgery. Front. Bioeng. Biotechnol..

[85] Karayilan M., Clamen L., Becker M.L. (2021). Polymeric Materials for Eye Surface and Intraocular Applications. Biomacromolecules.

[86] Ma Y.-C., Hsieh C.-T., Lin Y.-H., Dai C.-A., Li J.-H. (2021). Numerical Study of Customized Artificial Cornea Shape by Hydrogel Biomaterials on Imaging and Wavefront Aberration. Polymers.

[87] Shah R., Stodulka P., Skopalova K., Saha P. (2019). Dual Crosslinked Collagen/Chitosan Film for Potential Biomedical Applications. Polymers.

[88] Xu X., Liu Y., Fu W., Yao M., Ding Z., Xuan J., Li D., Wang S., Xia Y., Cao M. (2020). Poly(*N*-isopropylacrylamide)-Based Thermoresponsive Composite Hydrogels for Biomedical Applications. Polymers.

[89] Rodriguez-Galan A., Franco L., Puiggali J. (2010). Degradable Poly(ester amide)s for Biomedical Applications. Polymers.

[90] Roddier F., Roddier F. (1999). Adaptive Optics in Astronomy.

[91] Rimmele T.R., Marino J. (2011). Solar Adaptive Optics. Living Rev. Sol. Phys..

[92] Babcock H.W. (1953). The Possibility of Compensating Astronomical Seeing. Publ. Astron. Soc. Pac..

[93] Smirnov M.S. (1961). Measurement of the Wave Aberration of the Human Eye. Biofizika.

[94] Roorda A. (2011). Adaptive Optics for Studying Visual Function: A Comprehensive Review. J. Vis..

[95] Liang J., Williams D.R., Miller D.T. (1997). Supernormal Vision and High-Resolution Retinal Imaging through Adaptive Optics. J. Opt. Soc. Am. A.

[96] Dreher A.W., Bille J.F., Weinreb R.N. (1989). Active Optical Depth Resolution Improvement of the Laser Tomographic Scanner. Appl. Opt..

[97] Liang J. (2004). Wavefront Technology for Vision and Ophthalmology. Aberration-Free Refractive Surgery.

[98] Burns S.A., Elsner A.E., Sapoznik K.A., Warner R.L., Gast T.J. (2019). Adaptive Optics Imaging of the Human Retina. Prog. Retin. Eye Res..

[99] Roorda A., Romero-Borja F., Donnelly W., Hebert T., Queener H. (2002). Dynamic Imaging of Microscopic Retinal Features with the Adaptive Optics Scanning Laser Ophthalmoscope. Investig. Ophthalmol. Vis. Sci..

[100] Cheung C.Y., Ikram M.K., Chen C., Wong T.Y. (2017). Imaging Retina to Study Dementia and Stroke. Prog. Retin. Eye Res..

[101] Fernández E.J., Prieto P.M., Artal P. (2009). Binocular Adaptive Optics Visual Simulator. Opt. Lett..

[102] Chin S.S., Hampson K.M., Mallen E.A.H. (2008). Binocular Correlation of Ocular Aberration Dynamics. Opt. Express.

[103] Liang J. (2009). Methods and Devices for Refractive Treatments of Presbyopia. WO Patent.

[104] Bellucci R., Curatolo M.C. (2017). A New Extended Depth of Focus Intraocular Lens Based on Spherical Aberration. J. Refract. Surg..

[105] Spanò S.F., Anastasi E., Frison R., Mazzone M.G., Curatolo M.C. A new strategy in presbyopia correction: Mini well + mini well Proxa. Proceedings of the 39th Congress of the European Society of Cataract and Refractive Surgeons (ESCRS).

[106] Kohnen T. (2020). Nondiffractive Wavefront-Shaping Extended Range-of-Vision Intraocular Lens. J. Cataract Refract. Surg..

[107] Mencucci R., Cennamo M., Venturi D., Vignapiano R., Favuzza E. (2020). Visual Outcome, Optical Quality, and Patient Satisfaction with a New Monofocal IOL, Enhanced for Intermediate Vision: Preliminary Results. J. Cataract Refract. Surg..

[108] Domínguez-Vicent A., Esteve-Taboada J.J., del Águila-Carrasco A.J., Ferrer-Blasco T., Montés-Micó R. (2016). In Vitro Optical Quality Comparison between the Mini WELL Ready Progressive Multifocal and the TECNIS Symfony. Graefe’s Arch. Clin. Exp. Ophthalmol..

[109] Nowik K.E., Nowik K., Kanclerz P., Szaflik J.P. (2022). Clinical Performance of Extended Depth of Focus (EDOF) Intraocular Lenses—A Retrospective Comparative Study of Mini Well Ready and Symfony. Clin. Ophthalmol..

[110] Rosa N., de Bernardo M., Lanza M., Borrelli M., Fusco F., Flagiello A. (2008). Corneal Aberrations Before and after Photorefractive Keratectomy. J. Optom..

[111] Wallerstein A.A. (2003). Wavefront-Guided Refractive Surgery. Tech. Ophthalmol..

[112] Camellin M., Mosquera S.A. (2010). Simultaneous Aspheric Wavefront-Guided Transepithelial Photorefractive Keratectomy and Phototherapeutic Keratectomy to Correct Aberrations and Refractive Errors after Corneal Surgery. J. Cataract Refract. Surg..

[113] Smadja D., Reggiani-Mello G., Santhiago M.R., Krueger R.R. (2012). Wavefront Ablation Profiles in Refractive Surgery: Description, Results, and Limitations. J. Refract. Surg..

[114] Mrochen M., Donitzky C., Wüllner C., Löffler J. (2004). Wavefront-Optimized Ablation Profiles. J. Cataract Refract. Surg..

[115] Manns F., Ho A., Parel J.-M., Culbertson W. (2002). Ablation Profiles for Wavefront-Guided Correction of Myopia and Primary Spherical Aberration. J. Cataract Refract. Surg..

[116] Cogan D.G., Toussaint D., Kuwabara T. (1961). Retinal Vascular Patterns. Arch. Ophthalmol..

[117] McWhirter J.L., Noguchi H., Gompper G. (2009). Flow-Induced Clustering and Alignment of Vesicles and Red Blood Cells in Microcapillaries. Proc. Natl. Acad. Sci. USA.

[118] Zhong Z., Petrig B.L., Qi X., Burns S.A. (2008). In Vivo Measurement of Erythrocyte Velocity and Retinal Blood Flow Using Adaptive Optics Scanning Laser Ophthalmoscopy. Opt. Express.

[119] Guevara-Torres A., Joseph A., Schallek J.B. (2016). Label Free Measurement of Retinal Blood Cell Flux, Velocity, Hematocrit and Capillary Width in the Living Mouse Eye. Biomed. Opt. Express.

[120] Polans J., Cunefare D., Cole E., Keller B., Mettu P.S., Cousins S.W., Allingham M.J., Izatt J.A., Farsiu S. (2017). Enhanced Visualization of Peripheral Retinal Vasculature with Wavefront Sensorless Adaptive Optics Optical Coherence Tomography Angiography in Diabetic Patients. Opt. Lett..

[121] Chui T.Y.P., Zhong Z., Song H., Burns S.A. (2012). Foveal Avascular Zone and Its Relationship to Foveal Pit Shape. Optom. Vis. Sci..

[122] Tam J., Roorda A. (2011). Speed Quantification and Tracking of Moving Objects in Adaptive Optics Scanning Laser Ophthalmoscopy. J. Biomed. Opt..

[123] Salmon A.E., Cooper R.F., Langlo C.S., Baghaie A., Dubra A., Carroll J. (2017). An Automated Reference Frame Selection (ARFS) Algorithm for Cone Imaging with Adaptive Optics Scanning Light Ophthalmoscopy. Transl. Vis. Sci. Technol..

[124] Gofas-Salas E., Mecê P., Mugnier L., Bonnefois A.M., Petit C., Grieve K., Sahel J., Paques M., Meimon S. (2019). Near Infrared Adaptive Optics Flood Illumination Retinal Angiography. Biomed. Opt. Express.

[125] Piñero D.P., Cabezos I., López-Navarro A., de Fez D., Caballero M.T., Camps V.J. (2017). Intrasession Repeatability of Ocular Anatomical Measurements Obtained with a Multidiagnostic Device in Healthy Eyes. BMC Ophthalmol..

[126] Gordon-Shaag A., Piñero D.P., Kahloun C., Markov D., Parnes T., Gantz L., Shneor E. (2018). Validation of Refraction and Anterior Segment Parameters by a New Multi-Diagnostic Platform (VX120). J. Optom..

[127] Hénault F., Spang A., Feng Y., Schreiber L. (2020). Crossed-Sine Wavefront Sensor for Adaptive Optics, Metrology and Ophthalmology Applications. Eng. Res. Express.

[128] Pelzman C., Cho S.-Y. (2019). Wavefront Detection Using Curved Nanoscale Apertures. Appl. Phys. Lett..

[129] Teperik T.V., Archambault A., Marquier F., Greffet J.J. (2009). Huygens-Fresnel Principle for Surface Plasmons. Opt. Express.

[130] Luo X. (2018). Subwavelength Optical Engineering with Metasurface Waves. Adv. Opt. Mater..

[131] Zhang S., Wong C.L., Zeng S., Bi R., Tai K., Dholakia K., Olivo M. (2020). Metasurfaces for Biomedical Applications: Imaging and Sensing from a Nanophotonics Perspective. Nanophotonics.

[132] Decker M., Staude I., Falkner M., Dominguez J., Neshev D.N., Brener I., Pertsch T., Kivshar Y.S. (2015). High-Efficiency Dielectric Huygens’ Surfaces. Adv. Opt. Mater..

[133] Pfeiffer C., Grbic A. (2013). Metamaterial Huygens’ Surfaces: Tailoring Wave Fronts with Reflectionless Sheets. Phys. Rev. Lett..

[134] Chong K.E., Staude I., James A., Dominguez J., Liu S., Campione S., Subramania G.S., Luk T.S., Decker M., Neshev D.N. (2015). Polarization-Independent Silicon Metadevices for Efficient Optical Wavefront Control. Nano Lett..

[135] Shalaev M.I., Sun J., Tsukernik A., Pandey A., Nikolskiy K., Litchinitser N.M. (2015). High-Efficiency All-Dielectric Metasurfaces for Ultracompact Beam Manipulation in Transmission Mode. Nano Lett..

[136] Ballantine K.E., Ruostekoski J. (2021). Cooperative Optical Wavefront Engineering with Atomic Arrays. Nanophotonics.

[137] Ang M., Gatinel D., Reinstein D.Z., Mertens E., Alió del Barrio J.L., Alió J.L. (2021). Refractive Surgery beyond 2020. Eye.

[138] Ianchulev T., Hoffer K.J., Yoo S.H., Chang D.F., Breen M., Padrick T., Tran D.B. (2014). Intraoperative Refractive Biometry for Predicting Intraocular Lens Power Calculation after Prior Myopic Refractive Surgery. Ophthalmology.

[139] Spekreijse L.S., Bauer N.J.C., van den Biggelaar F.J.H.M., Simons R.W.P., Veldhuizen C.A., Berendschot T.T.J.M., Nuijts R.M.M.A. (2022). Predictive Accuracy of an Intraoperative Aberrometry Device for a New Monofocal Intraocular Lens. J. Cataract Refract. Surg..

[140] Gasparian S.A., Nassiri S., You H., Vercio A., Hwang F.S. (2022). Intraoperative Aberrometry Compared to Preoperative Barrett True-K Formula for Intraocular Lens Power Selection in Eyes with Prior Refractive Surgery. Sci. Rep..

[141] Krueger R.R., Shea W., Zhou Y., Osher R., Slade S.G., Chang D.F. (2013). Intraoperative, Real-Time Aberrometry During Refractive Cataract Surgery With a Sequentially Shifting Wavefront Device. J. Refract. Surg..

[142] Packer M. (2010). Effect of Intraoperative Aberrometry on the Rate of Postoperative Enhancement: Retrospective Study. J. Cataract Refract. Surg..

